# Evaluating vonoprazan and tegoprazan for gastroesophageal reflux disease treatment in Chinese Healthcare: an EVIDEM framework analysis

**DOI:** 10.1186/s12876-024-03297-6

**Published:** 2024-06-20

**Authors:** Chaojun Xue, Yuhan Du, Haotian Yang, Huixin Jin, Yue Zhao, Bingnan Ren, Zhanjun Dong

**Affiliations:** 1 Hebei General Hospital, Shijiazhuang, Hebei Province China; 2 Hebei Key Laboratory of Clinical Pharmacy, Shijiazhuang, Hebei Province China

**Keywords:** EVIDEM, Hospital-based health technology assessment, Vonoprazan, Tegoprazan

## Abstract

**Background:**

In Chinese healthcare settings, drug selection decisions are predominantly influenced by the Pharmacy & Therapeutics Committee (PTC). This study evaluates two recently introduced potassium-competitive acid blockers, vonoprazan (VPZ) and tegoprazan (TPZ), utilizing the Evidence and Value: Impact on DEcisionMaking (EVIDEM) framework.

**Methods:**

The study employed the 10th edition of EVIDEM, which includes a core model with five domains and 13 criteria. Two independent expert panels were involved: the PTC expert panel, tasked with assigning weights using a 5-point scale, defining scoring indicators, examining the evidence matrix, scoring, and decision-making; and the evidence matrix expert panel, responsible for conducting a systematic literature review, creating the evidence matrix, and evaluating the value contributions of VPZ and TPZ.

**Results:**

The analysis estimated the value contributions of VPZ and TPZ to be 0.59 and 0.54, respectively. The domain of ‘economic consequences of intervention’ showed the most significant variation in value contribution between the two drugs, followed by ‘comparative outcomes of intervention’ and ‘type of benefit of intervention’.

**Conclusion:**

Employing the EVIDEM framework, VPZ’s value contribution was found to be marginally superior to that of TPZ. The EVIDEM framework demonstrates potential for broader application in Chinese medical institutions.

**Supplementary Information:**

The online version contains supplementary material available at 10.1186/s12876-024-03297-6.

## Background

In Chinese medical institutions, the Pharmacy & Therapeutics Committee (PTC) plays a crucial role in selecting the most appropriate medications. This selection process is complex, involving multiple criteria, stakeholders, and underlying values [1]. Typically, pharmaceutical companies provide initial clinical evidence, which varies greatly in quantity and quality, thus presenting significant challenges for the PTC during the decision-making process [2]. Additionally, this process requires value judgments, often leading to disagreements stemming from differing views on the role of evidence and judgment in evidence-based medical decision-making [3]. Furthermore, the PTC is frequently pressed to make expedited decisions, highlighting the critical need for effective decision support tools.

The Evidence and Value: Impact on DEcisionMaking (EVIDEM) framework, devised by Goetghebeur et al., features a central model that includes five domains and 13 criteria, offering a structured approach to prioritization [4]. This framework enables decision-makers to articulate their values by assigning weights to various decision criteria, with the outcomes of these assessments translated into value estimates through a process of weighting and scoring. The mathematical components of the EVIDEM framework aim to enhance clarity, articulation, and the sharing of individual reasoning, thus bridging the gap between health technology assessment (HTA) and effective healthcare decision-making via multicriteria decision analysis (MCDA) [5]. Grounded in practical deliberation and decision-making, EVIDEM enhances the consistency, transparency, and legitimacy of decisions and is applicable across various healthcare settings [6]. This adaptable tool is specifically tailored to the intricate decision-making processes prevalent in medical institutions [1, 6, 7].

A variety of medications are available to manage gastroesophageal reflux disease (GERD), primarily focusing on suppressing acid production. Proton pump inhibitors (PPIs) are the standard treatment for GERD [8, 9]. However, the limitations of PPI therapy, such as PPI-refractory conditions, nocturnal acid breakthrough (NAB), and genetic polymorphism, cannot be overlooked [10]. Potassium-competitive acid blockers (P-CABs), such as vonoprazan (VPZ) and tegoprazan (TPZ), represent a novel class of acid suppressants that offer several advantages over PPIs, including rapid onset and prolonged acid suppression [11]. Despite their increasing availability, a comprehensive evaluation of the comparative effectiveness and overall value of VPZ and TPZ within the Chinese healthcare context has not yet been conducted. This study aims to employ the EVIDEM framework to assess these drugs comprehensively, with the expectation that the results will provide detailed insights into their value contributions, thereby supporting more informed and effective healthcare decision-making.

## Methods

### EVIDEM framework source

The study utilized the 10th edition of the EVIDEM framework, which has been developed over a decade through open-source contributions and is officially published [6]. This edition encompasses a core model with five domains and 13 criteria, as detailed in Table 1.

**Table 1:** The Evidence and Value: Impact on DEcisionMaking framework

Domains	Criteria	Type	Scoring Scale High→Low	Indicators
Need for intervention	Disease severity	Absolute	5 4 3 2 1 0 Very severe→Not severe	Morbidity, Progression, Quality of life
	Size of the affected population	Absolute	5 4 3 2 1 0 Common diseases→Very rare diseases	Disease incidence
	Unmet needs	Absolute	5 4 3 2 1 0 Many unmet needs→No unmet needs	Compliance, Response rate, Nocturnal acid breakthrough, Genetic polymorphism
Comparative outcomes of intervention	Comparative effectiveness	Relative	5 4 3 2 1 0–1 -2 -3 -4 -5 Much better than the comparator→No difference→Much worse than the comparator	Reflux esophagitis healing rate, Intragastric pH
	Comparative safety	Relative		Incidence of adverse effects
	Comparative patient-perceived health	Relative		Heartburn
Type of benefit of intervention	Type of preventive benefit	Absolute	5 4 3 2 1 0 Eradication→No preventive benefit	Preventive benefit
	Type of therapeutic benefit	Absolute	5 4 3 2 1 0 Cure→No therapeutic benefit	Therapeutic benefit
Economic consequences of intervention	Comparative cost consequences—costs of intervention	Relative	5 4 3 2 1 0–1 -2 -3 -4 -5 Substantial savings/Good affordability→No change in spending→Substantial additional expenditures/Bad affordability	Drug cost
	Comparative cost consequences—other medical costs	Relative		Pharmacoeconomic research in China
	Comparative cost consequences—non-medical costs	Relative		Pharmacoeconomic research in China
Knowledge about intervention	Quality of evidence	Absolute	5 4 3 2 1 0 Highly relevant and valid→Not relevant and/or invalid	Type of evidence, Level of evidence
	Expert consensus/clinical practice guidelines	Absolute	5 4 3 2 1 0 Strong recommendation for intervention above all other alternatives→Not recommended or invalid	Recommendation

### Criteria weighting and calculation methodology

In healthcare decision-making, weighting criteria are crucial to reflect the relative importance of each criterion within the overall assessment of healthcare interventions. This is crucial in MCDA, where various aspects like clinical effectiveness, cost-effectiveness, and patient-reported outcomes significantly contribute to the decision-making process [4, 5]. Weighting aids in prioritizing elements that may have greater impacts on healthcare outcomes or relevance to specific decision contexts, such as budget constraints. Additionally, the weights mirror the value system of the decision-makers, which may vary based on organizational, regional, or national policy goals.

The weights of 13 criteria were derived using MCDA, a structured approach that quantifies decision-making processes involving multiple criteria. The weights were derived from a consensus panel using the analytic hierarchy process (AHP). These evaluations were then numerically converted into weights, which were multiplied with the scores of each criterion for each healthcare intervention to calculate a composite score that guided the final decision-making process. This systematic and reproducible approach ensures transparency and alignment with the strategic health objectives of the organization or region.

### Expert panel design and duty

The study engaged two distinct expert groups: the PTC expert panel and the evidence matrix expert panel.

The selection of experts for the PTC panel was a highly meticulous process, aimed at covering a wide spectrum of expertise essential for a comprehensive evaluation of the drugs under consideration. This approach was also aligned with the legal requirements for the PTC in China. The panel consisted of fourteen members, and each member was deliberately chosen to represent various domains, including pharmacy, gastroenterology clinical medicine, nursing, hospital infection management, hospital health insurance, hospital quality control and healthcare management. This careful selection resulted in two experts from each of these areas. This diversity was of utmost importance to ensure a comprehensive assessment from multiple healthcare perspectives. By integrating a wide range of expertise, we ensured that every aspect of drug evaluation, from clinical effectiveness to practical implementation, was thoroughly considered. This inclusive approach significantly bolstered the strength and credibility of the study’s findings, facilitating a more holistic and well-rounded examination of the drugs in question.

The PTC experts conducted two offline meetings, fulfilling five key tasks. In the initial meeting, after thorough familiarization with the EVIDEM framework, the panel set weights for the 13 criteria on a 5-point scale (see Table 2) and developed evaluation indicators for these criteria (refer to Table 1).

**Table 2:** Weighting tool

Weight
5 High impact on the value of medicines (decisive)
4 Considerable impact on the value of medicines
3 Moderate impact on the value of medicines
2 Low impact on the value of medicines
1 Very low impact on the value of medicines (inessential)

Following extensive discussions and to minimize the influence of personal biases and potential conflicts of interest, the experts reached a consensus on how to score the relative indicators. Scores were to be determined based on results from studies comparing VPZ and TPZ with PPIs. If there was no significant difference between VPZ and TPZ when compared to PPIs, the score difference should not exceed 1 point. However, if a discernible distinction was observed between VPZ and TPZ in relation to PPIs, a score difference of more than 1 point would only be assigned after taking into consideration the overall benefits and drawbacks to the patient.

**Fig. 1:**
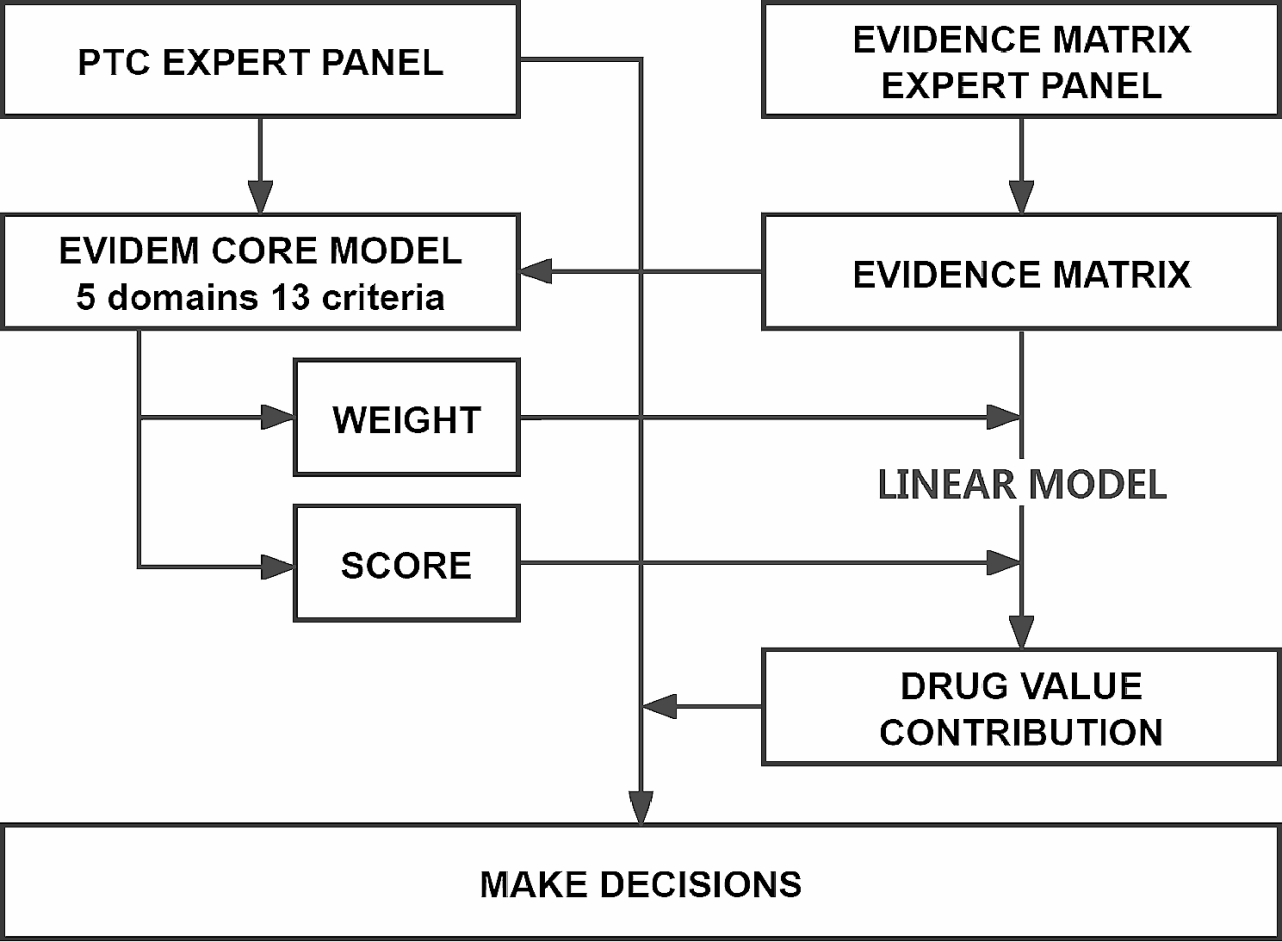
PTC decision-making process Notes: PTC, Pharmacy & Therapeutics Committee; EVIDEM, Evidence and Value: Impact on DEcisionMaking

After the completion of the evidence matrix, a second meeting was convened to review it. The PTC expert panel assigned scores to the 13 criteria based on the evidence matrix and then proceeded to make a drug selection decision. This decision was based on the value contributions of VPZ and TPZ as calculated by the evidence matrix expert panel. The decision-making process is illustrated in Fig. 1.

The evidence matrix expert panel, composed of three experts in evidence-based medicine, was assigned three primary tasks: (1) conducting a systematic literature review based on the indicators defined by the PTC expert panel, (2) creating the evidence matrix, and (3) calculating the value contributions of VPZ and TPZ.

### Literature review and evidence matrix

#### (1) Literature review process

A thorough literature review was undertaken to gather available evidence concerning the 13 criteria for VPZ and TPZ within the EVIDEM framework. Major biomedical databases such as PubMed, Embase, The Cochrane Library, and China Biology Medicine disc were searched, covering literature up to June 2023. The review was limited to Chinese and English language publications. The detailed search strategies for each database used in our study in the Supplementary file 1. Specific search terms were used in different domains.

- For the ‘need for intervention’, terms included vonoprazan, tegoprazan, gastroesophageal reflux disease, esophagitis, quality of life, epidemiology/prevalence/incidence, and mortality.
- For ‘comparative outcomes of intervention’ and ‘type of benefit of intervention’ domains, the terms included efficacy and safety related to the diseases.
- For pharmacoeconomic research specific to China, only studies conducted within the Chinese context were considered.
- In the ‘knowledge about intervention’ domain, keywords such as guidelines and clinical practice were included.

#### (2) Organizing the evidence

The evidence collected was systematically organized into an evidence matrix, which served as the foundational tool for subsequent evaluations. Each entry in the matrix was accompanied by a quality rating, derived from a standardized quality assessment tool. This tool helped in assessing the weight of each piece of evidence in the final decision-making process, ensuring a systematic and reproducible method that enhanced the transparency and accountability of the evaluative process.

#### (3) Minimizing bias

To ensure objectivity and minimize bias in the evaluation process, several strategies were employed.

- A double-blinded approach was used, where neither the members of the expert panels nor the study coordinators had access to identifiers that could link the evaluations to specific evaluators or studies being assessed. This blinding was maintained throughout the initial literature review and scoring phases.
- The criteria for selecting experts were rigorously defined to include a broad range of disciplines and perspectives, reducing potential bias towards particular outcomes.

#### (4) Addressing limitations in evidence strength

The evidence matrix expert panel implemented a robust methodological framework to address potential limitations posed by the availability and relevance of studies. This included.

- Expanded search criteria to capture a broader spectrum of relevant studies and incorporation of grey literature to supplement peer-reviewed articles.
- A consensus method where expert clinical judgment was integrated with the best available data for cases where evidence was limited or of low strength.
- Each study included in the evidence matrix was subject to rigorous critical appraisal focusing on methodological quality and relevance to the Chinese healthcare context, with transparent discussion of these factors in the evidence matrix.

### Data analysis

The overall estimated value contributions of VPZ and TPZ were determined using an additive linear model, as shown below:

1 $$V = \sum\limits_{x = 1}^n {Vx = } \sum\limits_{x = 1}^n {\left( {\frac{{Wx}}{{\sum {Wn} }}Sx} \right)}$$

where *V* is the total estimated value, *n* is the number of experts, *Vx* is the value contribution of criterion *x*, *Wx* is the weighting of criterion *x*, *ΣWn* is the sum of all weights, and *Sx* is the normalized score for each criterion *x* (*Sx* = score/5).

In the current analysis, the above additive linear model was employed due to its adeptness in integrating diverse criteria into a comprehensive value estimate. The simplicity and transparency of this model facilitate the straightforward interpretation of each criterion’s contribution to the final estimated value—an essential feature within the EVIDEM framework. As this framework is instrumental in guiding healthcare decision-making, it requires a model capable of representing the individual impact of varied factors without adding complexity that could diminish each criterion’s discernible importance.

The additive linear model is predicated on the assumption that the criteria are independent and contribute proportionally to the outcome. This presumption is congruent with the nature of multifaceted healthcare evaluations, where different factors—such as efficacy, safety, and economic implications—are considered not to interact in a manner that would significantly skew their combined influence on decision-making.

Our choice to implement an additive linear model is bolstered not merely by its theoretical aptness for the EVIDEM framework but also by its empirical substantiation in various peer-reviewed studies [12–14]. These publications provide valuable precedents that demonstrate the model’s capability to amalgamate various criteria into a coherent value estimate, upholding the principles of transparency and straightforward interpretation that are paramount for informed healthcare decision-making.

## Results

### Weights of 13 criteria

**Fig. 2:**
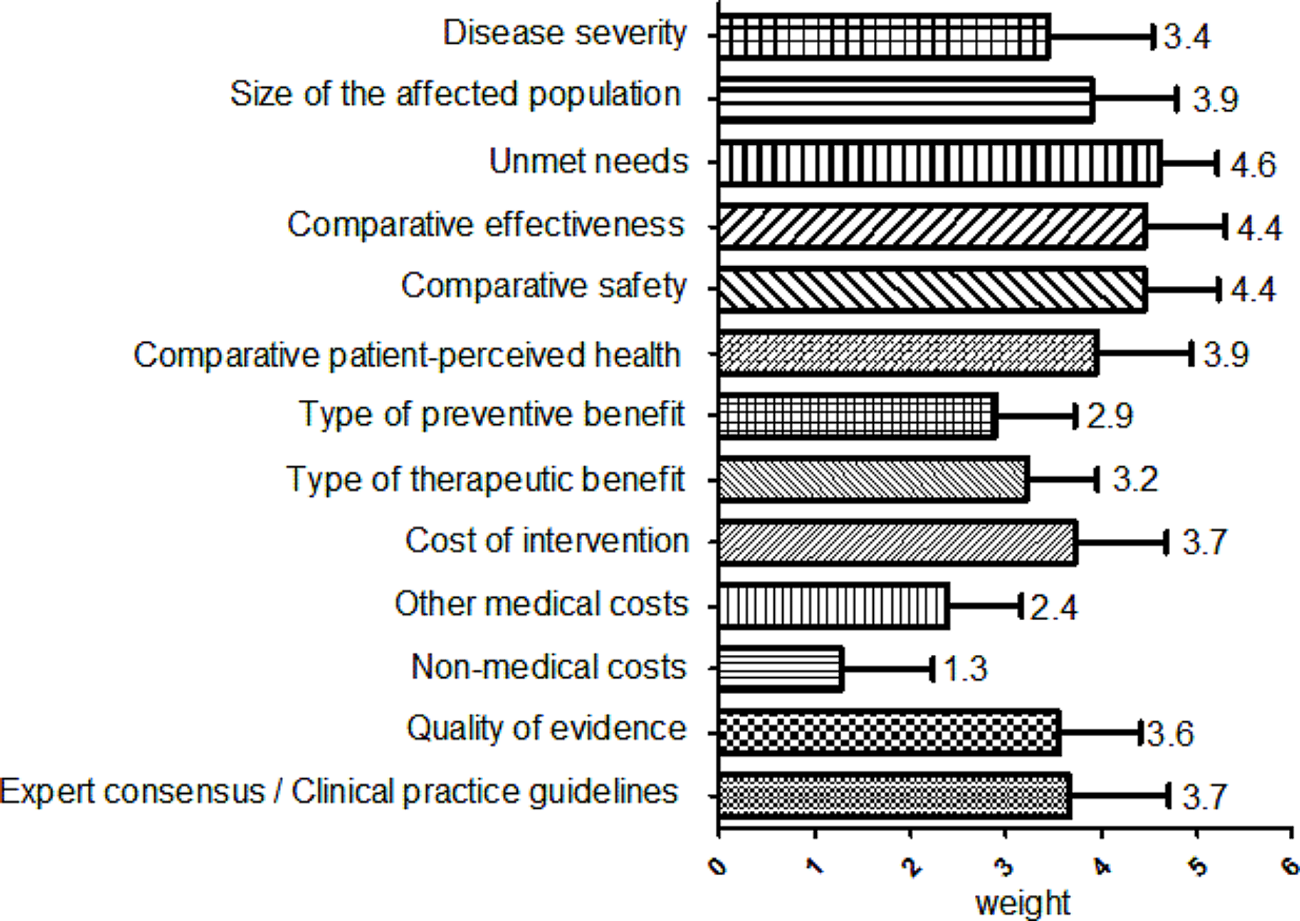
Weights for 13 criteria

The PTC expert panel established weights for each criterion in a single meeting, as depicted in Fig. 2. In the domain of ‘need for intervention’, ‘unmet needs’ received the highest average weight. Comparative effectiveness and safety were equally weighted. Experts in medical institutions focused on the therapeutic benefits of the drugs under evaluation. Within the domain of ‘economic consequences of intervention’, costs of intervention > other medical costs > non-medical costs. The quality of evidence criterion showed significant variability in weighting.

### Scores based on the evidence matrix

Table 3 summarizes the scores and key observations for all 13 criteria for VPZ and TPZ. The detailed evidence matrix is provided in Supplementary file 1.

**Table 3:** Scores and key comments of 13 criteria for VPZ and TPZ

Criteria	Scores (mean ± SD)	Comments
Disease severity	2.9 ± 0.6	Morbidity: There was no elevated risk of all-cause mortality associated with GERD. Progression: Most patients remained stable or showed improvement in their grade of esophagitis in 5 years. There was a positive association between reflux symptoms and the risk of esophageal adenocarcinoma. Quality of life: Respondents with GERD experienced heartburn, eating and drinking problems, sleep impairment, reduced work productivity, etc.
Size of the affected population	3.5 ± 1.1	Age-standardized prevalence of GERD in China is below 5%. Around 3,000 patients annually have been diagnosed with GERD in the study’s host hospital.
Unmet needs	4.2 ± 0.6	PPI response rate: 10–54% of patients with GERD symptoms failed to respond to a standard-dose PPI. PPI compliance: PPIs are used before meals and take 3–5 days to achieve a steady-state antisecretory effect. Nocturnal acid breakthrough: More than 70% of patients treated with PPIs had NAB. Genetic polymorphism: CYP2C19 genetic polymorphism causes significant inter-individual pharmacodynamic variability with PPI treatment.
Comparative effectiveness	VPZ 3.9 ± 1.1 TPZ 3.4 ± 1.0	VPZ and TPZ are non-inferior to PPIs for patients with GERD. VPZ may be more effective than PPIs for severe erosive esophagitis. Nocturnal pH ≥ 4 HTRs of VPZ and TPZ were greater than PPIs. 24-hour pH ≥ 4 HTRs of VPZ were greater than PPIs. The acid inhibitory effects of VPZ and TPZ are irrespective of the CYP2C19 genotype.
Comparative safety	VPZ 3.0 ± 0.6 TPZ 3.1 ± 0.5	Safety of VPZ and TPZ is comparable with PPIs.
Comparative patient-perceived health	VPZ 2.5 ± 1.0 TPZ 2.4 ± 1.2	There were no substantial differences in improvement of heartburn between VPZ and PPIs. Nocturnal heartburn improvement of VPZ and TPZ was greater than PPIs.
Type of preventive benefit	VPZ 3.6 ± 0.9 TPZ 3.4 ± 1.0	Long-term maintenance therapy and on-demand therapy with VPZ and TPZ can reduce the GERD recurrence rate.
Type of therapeutic benefit	VPZ 4.1 ± 0.9 TPZ 3.8 ± 1.1	The therapeutic target is to relieve symptoms, heal, prevent complications, and improve health-related quality of life. The therapeutic effect is mainly shown in improvement of reflux esophagitis healing rate, GerdQ/FSSG/GOS scores, heartburn symptoms, etc.
Costs of intervention	VPZ 0.9 ± 0.7 TPZ − 0.6 ± 0.5	In 2022, China’s per capita disposable income reached ¥36,883. The treatment phase takes 8 weeks, VPZ costs ¥558.88, and TPZ costs ¥626.08. The maintenance phase, VPZ costs ¥4.945 daily, TPZ costs ¥5.59 daily.
Other medical costs	VPZ 1.3 ± 0.9 TPZ 0	VPZ generates incremental QALYs at a lower cost compared with PPIs. TPZ retrieved no evidence.
Non-medical costs	VPZ 0 TPZ 0	Neither VPZ nor TPZ retrieved evidence.
Quality of evidence	2.4 ± 0.7	The quality of evidence on which the comparison data are based is highly relevant and valid. There is still a lack of direct comparisons between VPZ and TPZ.
Expert consensus/clinical practice guidelines	3.5 ± 1.0	The results of the current expert opinions and clinical practice guidelines clearly indicate that PPIs and P-CABs are the first-line treatment for GERD.

Notes: VPZ, vonoprazan; TPZ, tegoprazan; GERD, gastroesophageal reflux disease; NAB, nocturnal acid breakthrough; PPIs, proton-pump inhibitors; CYP2C19, cytochrome P450 2C19; pH ≥ 4 HTR, pH ≥ 4 holding time ratio; GerdQ, GERD questionnaire; FSSG, Frequency Scale for the Symptoms of GERD; GOS, Global Overall Symptom; QALYs, quality adjusted life years; P-CABs, potassium-competitive acid blockers

### Need for intervention domain

In the ‘need for intervention’ domain, the criterion of ‘disease severity’ received an average score of 2.9 ± 0.6, suggesting that the experts did not consider GERD to be particularly severe, despite its significant impact on patients’ quality of life, work, and emotional well-being [15].

The ‘size of the affected population’ criterion scored higher than ‘disease severity’. Although GERD incidence in China is lower compared to Europe and North America [16], the absolute number of patients remains substantial due to China’s large population. This was evidenced by approximately 3,000 diagnosed cases annually in the study’s host hospital. The criterion received an average score of 3.5 ± 1.1.

The ‘unmet needs’ criterion scored the highest among the 13 indicators with an average of 4.2 ± 0.6, indicating a significant unmet need for alternatives to PPI-based acid suppression therapy.

### Comparative outcomes of intervention domain

For the ‘comparative effectiveness’ criterion, key indicators included reflux esophagitis (RE) healing rate and pH ≥ 4 holding time ratios (HTRs). In the findings detailed in references [17–20], all patient groups demonstrated esophagitis healing rates exceeding 90% after 8 weeks of treatment with either P-CABs or PPIs. Specifically, healing rates for esophagitis treated with TPZ at dosages ranging from 50 to 100 mg and VPZ at dosages of 5–10 mg remained above this threshold. Notably, VPZ has shown potentially greater effectiveness, especially in severe cases of erosive esophagitis (EE) [17–19], where its efficacy surpasses that of traditional PPIs. In terms of nocturnal pH ≥ 4 HTRs, both VPZ and TPZ outperformed PPIs [21–25]. A separate study comparing TPZ and VPZ reported a treatment success rate of 66.0% for TPZ and 60.5% for VPZ, with a *p*-value of 0.30, indicating no statistically significant difference between the two [24]. During the 0–24 h period, pH ≥ 4 HTRs displayed variations. Three studies indicated significantly more favorable outcomes for the 20 mg VPZ compared to PPIs [21–23], whereas the results for the 50 mg TPZ group were similar to those of the PPIs [24, 25]. The experts concluded that the outcomes mentioned above favored VPZ over TPZ. On average, the scores given to VPZ and TPZ were 3.9 ± 1.1 and 3.4 ± 1.0, respectively.

Recent systematic reviews have revealed no significant differences in the occurrence of adverse events (AEs) among patients using VPZ compared to those on PPIs. Specifically, the incidences of any AEs (odds ratio [OR] = 0.96, *p* = 0.66), drug-related AEs (OR = 1.10, *p* = 0.44), serious AEs (OR = 1.14, *p* = 0.36), and AEs leading to drug discontinuation (OR = 1.09, *p* = 0.55) were comparable between VPZ and PPIs [26]. It is important to note that these figures indicate comparability in safety profiles rather than direct equivalence, highlighting the need for careful interpretation of these results in clinical contexts. Similarly, meta-analyses of TPZ, based on four clinical trials [20, 27–29], showed no significant differences in the incidences of any AEs (OR = 0.79, *p* = 0.22), drug-related AEs (OR = 0.82, *p* = 0.47), and serious AEs (OR = 2.25, *p* = 0.30) when compared to PPIs. These odds ratios suggest that TPZ and VPZ have safety profiles that are not significantly worse than those of PPIs, which supports their use under similar clinical conditions. The overall average scores for VPZ and TPZ on the ‘comparative safety’ criterion were 3.0 ± 0.6 and 3.1 ± 0.5, respectively, indicating no significant disparity in safety profiles according to experts.

Regarding heartburn symptoms, differences between VPZ and TPZ were noted. The percentages of 24-hour heartburn-free periods for VPZ 20 mg and esomeprazole 40 mg were 36.7% and 38.4%, respectively, without statistical significance [30]. Nevertheless, VPZ demonstrated superior efficacy in achieving complete nocturnal heartburn relief compared to lansoprazole (*p* < 0.01) [31]. In contrast, TPZ showed significantly higher complete resolution rates of heartburn than placebo [32], but its efficacy in terms of the time to the first nighttime heartburn-free interval and the percentage of nighttime heartburn-free days was similar to esomeprazole [28]. The overall average scores for VPZ and TPZ in the ‘comparative patient-perceived health’ criterion were 2.5 ± 1.0 and 2.4 ± 1.2, respectively.

### Type of benefit of intervention domain

In the ‘type of benefit of intervention’ domain, the ‘type of preventive benefit’ criterion received average scores of 3.6 ± 0.9 for VPZ and 3.4 ± 1.0 for TPZ. This reflects a consensus among experts that both VPZ and TPZ offer considerable preventive benefits, particularly in achieving high endoscopic remission rates. Data available for maintenance therapy shows VPZ’s effectiveness for up to 52 weeks [33] and TPZ’s for 24 weeks [29]. Notably, VPZ at a dose of 10 mg proved clinically effective in maintaining healed RE refractory to PPIs for 48 weeks [34].

The ‘type of therapeutic benefit’ criterion was scored 4.1 ± 0.9 for VPZ and 3.8 ± 1.1 for TPZ. These scores highlight the therapeutic advantages of both drugs, especially in improving the RE healing rate, as well as in enhancing GERD Questionnaire (GerdQ)/Frequency Scale for the Symptoms of GERD (FSSG)/Global Overall Symptom (GOS) scores, and alleviating heartburn symptoms. A key observation is that compared with PPIs, VPZ and TPZ demonstrated a faster onset of action and inhibited acid production irrespective of CYP2C19 genotype variations [24, 35]. Furthermore, VPZ was particularly effective for patients resistant to PPI-based RE treatment [36, 37].

### Economic consequences of intervention domain

The economic consequences were assessed based on three criteria, starting with the ‘costs of intervention’. In China, the treatment phase cost for VPZ is ¥558.88, while for TPZ, it is ¥626.08. These costs represent approximately 1.5% and 1.7%, respectively, of China’s per capita disposable income in 2022. Experts suggest that both drugs are relatively affordable for patients, with VPZ being slightly more economical. The average scores for VPZ and TPZ in this criterion were 0.9 ± 0.7 and − 0.6 ± 0.5, respectively.

A single study evaluated the cost-effectiveness of VPZ compared to PPIs in treating GERD patients in China. ‘Other medical costs’ mainly includes expenses for outpatient visits, endoscopies, and 24-hour pH monitoring [38]. This study concluded that VPZ is the dominant treatment option in terms of cost-effectiveness. Regarding ‘other medical costs’, VPZ and TPZ scored 1.3 ± 0.9 and 0, respectively.

No data were available to evaluate the ‘non-medical costs’, and hence the PTC expert panel did not assign scores to this criterion.

### Knowledge about intervention domain

All clinical trials included in this review were considered valid. However, there is a notable absence of direct comparisons between VPZ and TPZ. Consequently, the ‘quality of evidence’ criterion received a score of 2.4 ± 0.7, reflecting its relative lesser significance.

The final criterion appraised was ‘expert consensus/clinical practice guidelines’, which scored 3.5 ± 1.0. Despite PPIs remaining the predominant treatment for GERD, P-CABs have been increasingly recommended in clinical practice guidelines as a first-line treatment [39, 40]. Additionally, P-CABs have been recognized for their superior efficacy in controlling intragastric pH more effectively and rapidly than PPIs, particularly in cases of refractory GERD [10].

### Estimated value contribution of VPZ and TPZ

**Fig. 3:**
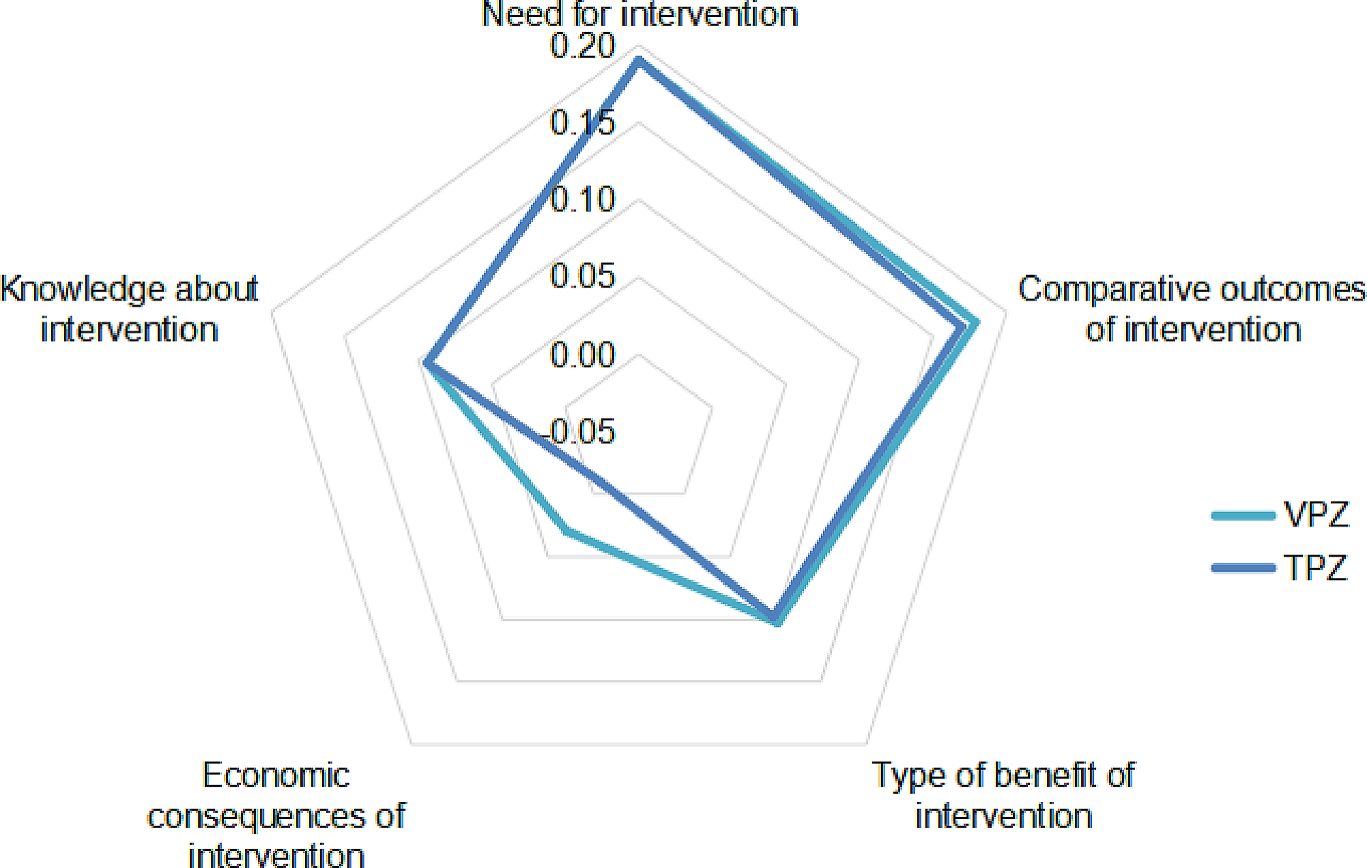
Estimated value contribution of vonoprazan (VPZ) and tegoprazan (TPZ)

The estimated value contributions for VPZ and TPZ were 0.59 and 0.54, respectively. Figure 3 illustrates the estimated value contributions across the five domains. The ‘economic consequences of intervention’ domain exhibited the most significant difference in value contribution between VPZ and TPZ, followed by the ‘comparative outcomes of intervention’ and ‘type of benefit of intervention’ domains.

## Discussion

In the context of GERD management, PPIs remain the primary drug treatment [8, 9]. Notably, up to 40% of GERD patients continue to experience symptoms despite PPI treatment [10]. Both PPIs and P-CABs target H^+^/K^+^-ATPase to inhibit acid secretion. As an alternative to PPIs, P-CABs have demonstrated their ability to suppress acid production by competitively inhibiting the gastric H^+^/K^+^-ATPase through K^+^ competition [11]. They also offer a prolonged inhibition with a gradual dissociation from the H^+^/K^+^-ATPase, resulting in long-lasting effects [41]. A clinical study with 24 patients with PPI-resistant RE reported a 87.5% success rate in treating esophageal mucosal breaks with 20 mg of VPZ [36]. Furthermore, NAB is a common occurrence, even when PPIs are administered twice daily. NAB is observed in over 70% of Helicobacter pylori-negative patients undergoing PPI therapy [42]. VPZ and TPZ have demonstrated more effective nighttime acid suppression compared to PPIs [21–25]. Additionally, substantial variation in pharmacodynamic responses among individuals is primarily attributed to CYP2C19 genetic polymorphism, significantly influencing the pharmacokinetics. Research has demonstrated that individuals classified as poor metabolizers based on their CYP2C19 enzyme activity are more prevalent in Asian populations (8–20%) when compared to Caucasians (3–5%) and African Americans (3–5%) [43]. Unlike PPIs, VPZ and TPZ’s efficacy in acid suppression is independent of the CYP2C19 phenotype [24, 35]. Finally, it is important to note that PPIs are susceptible to degradation in acidic conditions and typically require enteric coating. This leads to delayed absorption and onset of action [10]. However, clinical studies have indicated that VPZ and TPZ can be administered without the need to consider food intake [44, 45]. VPZ and TPZ address certain unmet needs in PPIs and provide a new option for the clinical management of GERD.

When it comes to evaluating relative indicators, our preference was to have data from trials directly comparing VPZ and TPZ. Unfortunately, only one such trial was available [24]. Consequently, for most of the relative criteria, we had to use PPIs as a reference to indirectly compare VPZ and TPZ. For example, in the assessment of the ‘comparative effectiveness’ criterion, VPZ and TPZ exhibited comparable results to PPIs in terms of RE healing rate and nocturnal pH ≥ 4 HTRs [17–25]. Nevertheless, VPZ demonstrated higher efficacy in severe EE cases [17–20] and more favorable 0–24 h pH ≥ 4 HTRs compared to PPIs [21–23]. TPZ exhibited acid suppression effects that were not significantly longer at night compared to VPZ but were greater than those observed with esomeprazole [24]. Furthermore [25], demonstrated that up to 12 h after evening dosing, TPZ showed a stronger acid-suppressive effect than dexlansoprazole, although both medications presented comparable acid-suppressive effects 12 h post-dosing. Given these considerations, it is more likely that patients will benefit from VPZ in terms of efficacy, resulting in scores of 3.9 ± 1.1 and 3.4 ± 1.0 for VPZ and TPZ, respectively.

The method we employed for scoring treatment effectiveness through expert consensus and comparative study results offers several advantages, including reducing individual biases, grounding decisions in empirical evidence, and ensuring flexibility within a structured framework that prioritizes patient outcomes. However, this method also presents challenges, such as potential subjectivity in interpreting “significant” differences, reliance on the availability and quality of comparative studies, and possible oversimplification of complex drug efficacy data. Additionally, the consensus process can be time-consuming and may be influenced by dominant personalities within the group, potentially skewing results. While this approach is patient-centric and evidence-based, it requires careful implementation to address these limitations and ensure that scoring accurately reflects nuanced clinical realities. This nuanced understanding is crucial, especially when indirect comparisons are necessitated by the scarcity of direct comparative trials.

This study clarified the complex process involved in selecting VPZ and TPZ for GERD treatment. It marked the first instance of our medical institution employing the EVIDEM framework to support drug selection decisions, an approach that has only been reported once before in China [1]. The EVIDEM framework serves as a bridge between evidence and decision-making. All PTC experts expressed confidence in the evaluation results and looked forward to its continued use. They also deliberated on the evidence matrix and the potential implications of the evaluation results for clinical practice.

The experts unanimously recognized that respecting patients’ wishes and values is a fundamental prerequisite for prescribing. Taking the patient’s perspective into account is especially important when prescribing P-CABs for patients who are PPI-refractory [36], experience NAB [21–25], possess specific CYP2C19 genotypes indicative of rapid metabolism [24, 35], or have difficulties adhering to premeal dosing [44, 45]. For example, VPZ should be prescribed for patients with EE and for those who prioritize economic considerations. Prioritizing the patient’s viewpoint reflects the principles of respecting patient autonomy and dignity, both of which are integral aspects of the healthcare system, as emphasized in articulated principles [46].

The Grading of Recommendations Assessment, Development, and Evaluation (GRADE) Working Group has also acknowledged the importance of incorporating patients’ values and wishes into clinical evidence-based guidelines [47]. Furthermore, the PTC experts explored the possibility of introducing a minimum of two patient representatives, a practice that has not been previously reported in drug selection decisions within Chinese medical institutions.

Although this study lays an important foundation for the PTC in making drug selection decisions, it exhibits several limitations. Primarily, the absence of direct clinical trials comparing VPZ and TPZ necessitated the use of PPIs as the comparator. This approach permits only indirect comparisons, thereby diminishing the ‘quality of evidence’, which was scored at 2.4 ± 0.7. Furthermore, by focusing exclusively on pharmacoeconomic studies conducted in China, the research encounters two significant challenges: a limited evidence base and a narrow view of the broader pharmacoeconomic discussion. The PTC expert panel also did not evaluate the ‘non-medical costs’. The absence of an expert panel for monitoring and maintaining the quality of evidence is an aspect that needs improvement in future research. Finally, in December 2023, alongside VPZ and TPZ, keverprazan (KPZ) finalized an agreement with the China Healthcare Security Administration. An expert panel is scheduled to conduct an EVIDEM framework analysis on all three P-CABs to evaluate their respective efficacies and safety profiles comprehensively.

## Conclusion

Utilizing the EVIDEM framework, this study estimates that the value contribution of VPZ is marginally higher than that of TPZ. The EVIDEM decision framework effectively converts evidence-based information into a quantifiable drug value assessment. This process facilitates the comparison and ranking of drugs, aiding decision-makers in making informed, scientific choices. Given its efficacy, the EVIDEM framework holds considerable promise for adoption in medical institutions.

### Electronic supplementary material

Below is the link to the electronic supplementary material.

Supplementary Material 1

## Data Availability

Data is provided within the manuscript or supplementary information files.

## Abbreviations

AEs: Adverse events
CYP2C19: Cytochrome P450 2C19
EVIDEM: Evidence and value: impact on decisionmaking
EE: Erosive esophagitis
FSSG: Symptoms of gastroesophageal reflux disease
GERD: Gastroesophageal reflux disease
GerdQ: Gastroesophageal reflux disease Questionnaire
GOS: Global Overall Symptom
HTA: Health technology assessment
HTRs: Holding time ratios
MCDA: Multicriteria decision analysis
NAB: Nocturnal acid breakthrough
OR: Odds ratio
P-CABs: Potassium-competitive acid blockers
PTC: Pharmacy & therapeutics committee
PPIs: Proton pump inhibitors
RE: Reflux esophagitis
TPZ: Tegoprazan
VPZ: Vonoprazan
