# Supplementary material for: Evaluating vonoprazan and tegoprazan for gastroesophageal reflux disease treatment in Chinese Healthcare: an EVIDEM framework analysis
Source: BMC Gastroenterol. 2024 Jun 20;24:208. doi: 10.1186/s12876-024-03297-6 (PMC11188247; doi:10.1186/s12876-024-03297-6)
Supplement: Supplementary file 1 — Supplementary Material 1 [file 12876_2024_3297_MOESM1_ESM.docx]

**Evaluating Vonoprazan and Tegoprazan for Gastroesophageal Reflux Disease Treatment in Chinese Healthcare: An EVIDEM Framework Analysis**

Chaojun. Xue^1,2^, Yuhan Du^1^, Haotian. Yang^1,2^, Huixin Jin^1,2^, Yue Zhao^1,2^, Bingnan Ren^1,2^, Zhanjun Dong^[[1]](#footnote-0),2^*

**Introduction**

Multiple drugs that suppress acid are available to treat gastroesophageal reflux disease (GERD), each with distinct mechanisms of action. The most potent medications inhibit the gastric H^+^/K^+^-ATPase to block the ultimate common pathway for acid secretion, either by covalent binding in the case of proton pump inhibitors (PPIs) or by reversible inhibition in the case of potassium competitive acid blockers (P-CABs). PPIs dominate this class of drugs, while P-CABs like vonoprazan (VPZ) and tegoprazan (TPZ) have more recently gained approval in China. The objective of this study was to assess the effectiveness of VPZ and TPZ within the Evidence and Value: Impact on DecisionMaking (EVIDEM) framework to determine the most suitable medication for use in Chinese medical institutions. To streamline the analysis and avoid excessive complexity, we chose PPIs as the comparator for this EVIDEM evaluation.

**SUPPLEMENTARY FILE**

[1. Need for intervention (Absolute Criterion)](#_Toc32448)

[1.1 Disease severity](#_Toc22302)

[1.2 Size of the affected population](#_Toc11968)

[1.3 Unmet needs](#_Toc29462)

[2. Comparative outcomes of intervention (Relative Criterion)](#_Toc15381)

[2.1 Comparative effectiveness](#_Toc30724)

[2.2 Comparative safety](#_Toc13476)

[2.3 Comparative patient-perceived health](#_Toc19948)

[3. Type of benefit of intervention (Absolute Criterion)](#_Toc29516)

[3.1 Type of preventive benefit](#_Toc21363)

[3.2 Type of therapeutic benefit](#_Toc5719)

[4. Economic consequences of intervention (Relative Criterion)](#_Toc26382)

[4.1 Comparative cost consequences—costs of intervention](#_Toc7797)

[4.2 Comparative cost consequences—other medical costs](#_Toc23182)

[4.3 Comparative cost consequences—non-medical costs](#_Toc8838)

[5. Knowledge about intervention (Absolute Criterion)](#_Toc11334)

[5.1 Quality of evidence](#_Toc9918)

[5.2 Expert consensus/clinical practice guidelines](#_Toc31259)

**1. Need for intervention (Absolute Criterion)**

## **1.1 Disease severity**

**Description of the disease**

- GERD is a medical condition that develops when the reflux of stomach contents causes troublesome symptoms and/or complications[1].
- GERD can manifest in three different phenotypic presentations. The most common presentation is non-erosive reflux disease (NERD), followed by erosive esophagitis (EE), and Barrett's esophagus (BE)[1].
- In general, signs and symptoms attributed to GERD are classified as oesophageal or extra-oesophageal. Heartburn is considered the characteristic symptom of GERD[1].
- The most severe potential consequence of GERD is esophageal adenocarcinoma (EAC). Patients with severe reflux symptoms (>3 times weekly for >5 years) have a 16-fold increased risk of EAC[2].

**Diagnosis**

- GERD is usually diagnosed based on either the response of suspected reflux-related symptoms to empiric acid-suppressive therapy or the objective findings of diagnostic tests[3-5].
- The presence of esophagitis on endoscopy as measured by the Los Angeles (LA) Classification (Grade A to Grade D with increasing severity) is sufficient to confirm GERD[3-5].
- For those with minimal changes, narrow-band imaging, oesophageal biopsy, pH testing (wireless or intranasal) or mucosal integrity testing may be helpful[3-5].
- The Reflux Disease Questionnaire (RDQ) and the GERD Questionnaire (GerdQ) serve as valuable research instruments for evaluating GERD[3-5].

**Mortality**

- The risks of all-cause mortality (HR = 1.00, 95% CI: 0.94–1.07) and cancer-specific mortality (HR = 0.99, 95% CI: 0.89–1.10) did not increase with GERD[6].
- People with BE and GRED have higher mortality rates than the general population, hazard ratios for all causes of death were 1.37(1.12–1.66) for the BE, 1.16(1.02–1.32) for the esophagitis, and 1.16(1.01–1.33) for the reflux cohorts[7].
- Prevalence of BE was significantly higher in men, both for endoscopically suspected (OR = 2.1, 95% CI: 1.6–2.8) and histologically confirmed BE (OR = 2.3, 95% CI: 1.7–3.2)[8].

**Progression**

- Most patients remained stable or showed improvement in their grade of esophagitis in 5 years[9, 10].
- In 5 years, 24.7% of the NERD patients, 1% of the LA grade A/B patients progressed to more severe grades[10].
- In 5 years, 5.9% of the NERD patients, 12.1% of the LA grade A/B patients and 19.7% of the LA grade C/D patients had endoscopic or confirmed BE[10].
- Annual progression rates for NERD developing EE ranged from 0% to 30%. About 1–22% of patients with mild EE developed more severe inflammation annually, while 1–13% of patients with EE developed BE annually[9].

**Quality of life**

- Respondents with GERD experienced eating and drinking problems (47%), sleep impairment (32%), and reduced work productivity (32%)[11].

## **1.2 Size of the affected population**

- In 2019, there were 783.95 million cases of GERD globally[12].
- Between 1990 and 2019, the total number of prevalent cases, incident cases, and years lived with disability increased by 77.53%, 74.79%, and 77.19%, respectively[13].
- The lowest age-standardized prevalence rate, age-standardized incidence rate, and age-standardized years of life lived with disability rate were found in China [4509.32(3899.11–5133.17) per 100,000 population, 1841.66(1607.09–2133.51) per 100,000 population, and 34.94(17.73–63.02) per 100,000 population, respectively][13].
- The prevalence of GERD varies with age. 14.0%(95% CI: 9.9%–18.7%) in those younger than 50 years and 17.3%(95% CI: 13.3%–21.7%) in those 50 years and older, for an odds ratio (OR) of 1.32(95% CI: 1.12–1.54)[14].

## **1.3 Unmet needs**

**Effectiveness**

- **Proton pump inhibitor-refractory GERD:** 10%–54% of patients with GERD symptoms failed to respond adequately, either partially or completely, to a standard-dose PPI[15-18].
- **Nocturnal acid breakthrough (NAB):** NAB is defined as the presence of at least 60 continuous minutes of intragastric pH<4 during the overnight period (22:00–06:00 h) in patients taking a PPI twice-daily before meals. NAB symptoms are the most common presentation of refractory reflux symptoms. NAB was shown to occur in more than 70% of patients on the PPI therapy[5].
- **Genetic polymorphism:** There is a significant inter-individual pharmacodynamic variability due to cytochrome P450 (CYP) 2C19 genetic polymorphism and the associated effects on pharmacokinetics. CYP2C19 rapid metabolizer genotype is a risk factor for PPI refractoriness in GERD patients with esophagitis. Efficacy rates of PPIs varied significantly between CYP2C19 genotypes (rapid metabolizers [RMs], 52.2%; intermediate metabolizers [IMs], 56.7%; poor metabolizers [PMs], 61.3%; *p* = 0.047)[19].

**Safety**

- Emerging research suggests that long-term PPI treatment might be associated with adverse events or complications, including kidney diseases, certain infections, osteoporosis, and gastric cancer[20].

**Compliance**

- PPIs are susceptible to acid degradation, requiring the use of enteric coating, which in turn results in delayed absorption and a slower onset of action[5].
- PPIs take 3–5 days to achieve a full, steady-state antisecretory effect[5].
- PPIs need to be taken half an hour to an hour before meals for better results[5].

**2. Comparative outcomes of intervention (Relative Criterion)**

## **2.1 Comparative effectiveness**

**Reflux esophagitis healing rate**

Supplementary Table 1 Reflux esophagitis healing rate of vonoprazan and tegoprazan

| Author(s), year | Country | Study design | Subjects | Treatment (sample size) | Treatment duration | Healing rates (%) |
| --- | --- | --- | --- | --- | --- | --- |
| Ashida et al., 2015[21] | Japan | RCT | EE patients | LPZ 30 mg, QD(140)  VPZ 5 mg, QD(148)  VPZ 10 mg, QD(145)  VPZ 20 mg, QD(154)  VPZ 40 mg, QD(146) | 8 weeks | Week 2: VPZ 5, 10, 20, 40 mg/86.0%, 93.2%, 93.8%, 94.8% vs. LPZ 88.6%  Week 4: VPZ 5, 10, 20, 40 mg/92.3%, 92.5%, 94.4%, 97.0% vs. LPZ 93.2%  Week 8: VPZ 5, 10, 20, 40 mg/96.5%, 95.5%, 96.5%, 97.0% vs. LPZ 95.5% |
| Ashida et al., 2015[22] | Japan | RCT | EE patients | VPZ 20 mg, QD(207)  LPZ 30 mg, QD(198) | 8 weeks | Week 2: VPZ 90.7% vs. LPZ 81.9%  Week 4: VPZ 96.6% vs. LPZ 92.5%  Week 8: VPZ 99.0% vs. LPZ 95.5% |
| Xiao et al., 2019[23] | China, South Korea, Malaysia | RCT | EE patients | VPZ 20 mg, QD(230)  LPZ 30 mg, QD(224) | 8 weeks | Week 2: VPZ 75.0% vs. LPZ 67.8%  Week 4: VPZ 85.3% vs. LPZ 83.5%  Week 8: VPZ 92.4% vs. LPZ 91.3% |
| Lee et al., 2019[24] | South Korea | RCT | EE patients | TPZ 50 mg, QD(92)  TPZ 100 mg, QD(91)  EPZ 40 mg, QD(22) | 8 weeks | Week 4: TPZ 50, 100 mg/91.3%, 93.4% vs. EPZ 94.3%  Week 8: TPZ 50, 100 mg/98.9%, 98.9% vs. EPZ 98.9% |

Notes: EE, erosive oesophagitis; RCT, randomized controlled trial; VPZ, vonoprazan; LPZ, lansoprazole; TPZ, tegoprazan; EPZ, esomeprazole.

**pH≥4 holding time ratios**

Supplementary Table 2 pH≥4 holding time ratios of vonoprazan and tegoprazan

| Author(s), year | Country | Study design | Subjects | Treatment | Outcomes |
| --- | --- | --- | --- | --- | --- |
| Takeuchi et al., 2020[25] | Japan | Multicenter, randomized, open-label, two-period, crossover study | Healthy adults | Cohort A: RPZ 20 mg/day (RPZ 20) vs. VPZ 20 mg/day (VPZ 20)  Cohort B: VPZ 20 vs. RPZ 40  Cohort C: VPZ 10 vs. RPZ 20  Each cohort consists of 10 people  Period 1 for 7 days, wash out at least 7 days, and period 2 for 7 days. | 24 h pH≥4 HTRs:  VPZ 10, 69.9%; VPZ 20, 88.4%–95%  RPZ 20, 53.8%–69.5%; RPZ 40, 74.5%  Nocturnal pH≥4 HTRs:  VPZ 10, 54.0%; VPZ 20, 70.7%–91.5%  RPZ 20, 57.1%–59.8%; RPZ 40, 73.2% |
| Sakurai et al., 2015[26] | Japan | Randomized, open-label, two-period, crossover study | Healthy adults | Cohort A: VPZ 20 mg/day (VPZ 20) vs. EPZ 20 mg/day (EPZ 20)  Cohort B: VPZ 20 vs. RPZ 20 mg/day (RPZ 20)  Each cohort consists of 10 people  Period 1 for 7 days, wash out at least 7 days, and period 2 for 7 days.  Drugs were administered at time 0 (as a rule, 9:00 AM). | 0–24 h pH≥4 HTRs:  VPZ 20 vs. EPZ 20 = 85.8% vs. 61.2%  VPZ 20 vs. RPZ 20 = 93.8% vs. 65.1%  12–24 h pH≥4 HTRs:  VPZ 20 vs. EPZ 20 = 75.2% vs. 44.8%  VPZ 20 vs. RPZ 20 = 88.8% vs. 54.1% |
| Laine et al., 2022[27] | USA | Randomized, open-label, two-period, crossover study | Healthy adults | VPZ 20 mg (VPZ20, 40 people) and LPZ 30 mg (LPZ30, 41 people) were administered orally once daily for a 7-day period, separated by a washout interval of ≥7 days. | 24 h pH≥4 HTRs:  VPZ 20 vs. LPZ 30 = 87.8% vs. 42.3% |
| Yang et al., 2022[28] | Korea | Randomized, open-label, single-dose, 3-treatment, 3-period, 6-sequence crossover design | Healthy adults | TPZ 50 mg (TPZ50), VPZ 20 mg (VPZ20), and EPZ 40 (EPZ40) were administered orally once daily for a 7-day period, separated by a washout interval of ≥7 days. Each group consists of 10 people. | Nocturnal pH≥4 HTRs:  TPZ 50 vs. VPZ 20 vs. EPZ 40 = 66.0% vs. 60.5% vs. 36.1% |
| Han et al., 2022[29] | Korea | Randomized, open-label, single-dose, balanced incomplete block crossover study | Healthy adults | The 24 subjects were assigned to receive DPZ 60 mg (DPZ60) or TPZ 50, 100, or 200 mg (TPZ50, 100, 200) orally during each of two administration periods, separated by a washout interval of 7 to 10 days. | 0–24 h pH≥4 HTRs:  TPZ 50, 100, 200 = 58.55%, 70.07%, 81.73%  DPZ 60 = 60.55%  Nocturnal pH≥4 HTRs:  TPZ 50, 100, 200 = 65.11%, 65.61%, 78.15%  DPZ 60 = 37.78% |

Notes: VPZ, vonoprazan; RPZ, rabeprazole; LPZ, lansoprazole; TPZ, tegoprazan; EPZ, esomeprazole; DPZ, dexlansoprazole; pH≥4 HTR, pH≥4 holding time ratio.

**2.2 Comparative safety**

**VPZ vs. PPIs**

- A large number of systematic reviews have reported safety outcomes for VPZ vs. PPIs[30-32].
- The pooled incidences of any AEs, drug-related AEs, serious AEs, and AEs leading to drug discontinuation were 20%, 7%, 1%, and 1%, respectively.
- The incidences of any AEs (OR = 0.96, *p* = 0.66), drug-related AEs (OR = 1.10, *p* = 0.44), serious AEs (OR = 1.14, *p* = 0.36), and AEs leading to drug discontinuation (OR = 1.09, *p* = 0.55) were not significantly different between patients taking VPZ and PPIs.
- Diarrhea or loose stool analysis showed that there was a statistically significant difference between VPZ and PPI treatment (95% CI = 0.661–0.966, *p* = 0.021).
- The VPZ therapy was effective as both initial and maintenance treatment across three patient groups: reflux esophagitis (RE), NERD, and PPI-resistant GERD. Notably, in the PPI-resistant GERD group, VPZ therapy significantly alleviated heartburn symptoms and enhanced patient satisfaction[33].

**TPZ vs. PPIs**

- A total of four clinical studies reported the adverse event rate of TPZ vs. PPIs[24, 34-36].
- The pooled incidences of any AEs, drug-related AEs, and serious AEs were 23.6%, 10.8%, and 1.5%, respectively.
- The incidences of any AEs (OR = 0.79, *p* = 0.22), drug-related AEs (OR = 0.82, *p* = 0.47), and serious AEs (OR = 2.25, *p* = 0.30), were not significantly different between patients taking TPZ and PPIs.
- Drug-related AEs were less frequent in the TPZ 25 mg group (12.7%, 22/173 patients) than in the lansoprazole 15 mg group (21.3%, 37/174 patients) (*p* = 0.0341)[36].
- Once daily administration of TPZ 50 or 100 mg showed non-inferior efficacy in healing EE and tolerability to that of esomeprazole 40 mg[24].

## **2.3 Comparative patient-perceived health**

**Heartburn**

Supplementary Table 3 Heartburn-free ratio of vonoprazan and tegoprazan

| Author(s), year | Country | Study design | Subjects | Treatment (sample size) | Treatment duration | Endpoint | Outcomes |
| --- | --- | --- | --- | --- | --- | --- | --- |
| Tackl et al., 2022[37] | Europe | RCT | GERD patients | EPZ 40 mg, QD(86)  VPZ 20 mg, QD(85)  VPZ 40 mg, QD(85) | 4 weeks | Percentages of heartburn-free | EPZ 40 = 36.5%  VPZ 20 = 36.7%  VPZ 40 = 38.4% |
| Oshima et al., 2019[38] | Japan | RCT | GERD patients | LPZ 30 mg, QD(16)  VPZ 20 mg, QD(16) | 2 weeks | Complete heartburn relief | VPZ vs. LPZ  HR: 3.58, 95% CI: 1.16–11.08 |
| Kinoshita et al., 2019[39] | Japan | RCT | NERD patients | Placebo, QD(232)  VPZ 10 mg, QD(244) | 4 weeks | Proportion of days without heartburn | Placebo = 61.4%  VPZ 10 = 73.6% |
| Kim et al., 2021[40] | Korea | RCT | NERD patients | Placebo, QD(99)  TPZ 50 mg, QD(106)  TPZ 100 mg, QD(99) | 4 weeks | Proportion of patients with complete resolution of heartburn | Placebo = 56.7%  TPZ 50 = 67.6%  TPZ 100 = 66.5% |
| Kim et al., 2023[41] | Korea | RCT | GERD patients | EPZ 40 mg, QD(21)  TPZ 50 mg, QD(19) | 2 weeks | Percentage of nighttime heartburn-free days | EPZ 40 = 42.8±31.3%  TPZ 50 = 52.4±28.7% |

Notes: GERD, gastroesophageal reflux disease; NERD, non-erosive reflux disease; RCT, randomized controlled trial; VPZ, vonoprazan; LPZ, lansoprazole; TPZ, tegoprazan; EPZ, esomeprazole.

**3. Type of benefit of intervention (Absolute Criterion)**

## **3.1 Type of preventive benefit**

- NERD patients and those with cured EE can benefit from the prophylactic use of VPZ and TPZ. Prophylactic use of VPZ and TPZ can be accomplished in two ways: long-term maintenance therapy and on-demand therapy[41].
- VPZ: Data for the maintenance therapy with VPZ have been available for up to 52 weeks[42]. The maintenance therapy with VPZ on a daily basis is effective for RE and those who are resistant to the standard dose of PPIs[43]. The preventive benefit of patients is mainly manifested in the reduction of GERD recurrence rate. The following factors may also affect the recurrence rate: patients who were: aged < 65 years; of either gender; nonsmokers; had any LA classification grade; CYP2C19 extensive metabolizers; or Helicobacter pylori-negative. Because the acid clearance capability of individuals with mild RE is nearly comparable to that of healthy subjects, effectively managing symptoms that negatively impact their quality of life becomes paramount in the treatment of mild RE. The optimal treatment for mild RE is on-demand therapy. When using the on-demand therapy with a 20 mg dose of VPZ, remission was successfully sustained in 86% of all patients with mild RE[44]. VPZ 10 mg was clinically effective for the maintenance of healed RE refractory to PPIs for 48 weeks[45].
- TPZ: Patients who had confirmed healing of EE through endoscopy received a once-daily dose of 25 mg of TPZ for a duration of up to 24 weeks. After 24 weeks, the endoscopic remission rate for TPZ was found to be 90.6%[36].

**3.2 Type of therapeutic benefit**

- The success rate of treating GERD largely depends on the ability to sustain an intragastric pH level of 4 or higher. Previous trials (Supplementary Table 2) have demonstrated that pH≥4 HTRs of VPZ and TPZ were significantly higher than that of PPIs daily. VPZ and TPZ produced more rapid, potent, and well sustained nighttime acid suppression than PPIs. Compared with PPI, VPZ and TPZ inhibited acid production irrespective of CYP2C19 genotype[28, 46]. VPZ was effective for most patients with PPI-resistant RE[47].
- The therapeutic target for EE is to relieve symptoms, heal and maintain remission of EE, prevent complications, and improve health-related quality of life. Initial treatment and maintenance phases should be applied in the treatment for EE[48]. Currently, P-CABs have shown good therapeutic effects in both phases of EE treatment. The therapeutic effect is mainly shown in improvement of reflux esophagitis healing rate, GerdQ/Frequency Scale for the Symptoms of GERD (FSSG)/Global Overall Symptom (GOS) scores, heartburn symptoms, etc.

**4. Economic consequences of intervention (Relative Criterion)**

## **4.1 Comparative cost consequences—costs of intervention**

- In 2022, China's per capita disposable income reached ¥36,883.
- VPZ fumarate tablets, 20 mg per tablet, 7 tablets per box, costs ¥69.3 per box in China. The treatment phase takes 8 weeks, 20 mg per day, and costs ¥558.88. The maintenance phase takes 10 mg per day, costs ¥4.945 per day.
- TPZ tablets, 50 mg per tablet, 7 tablets per box, costs ¥78.26 per box in China. The treatment phase takes 8 weeks, 50 mg per day, and costs ¥626.08. The maintenance phase takes 250 mg per day, and costs ¥5.59 per day.

## **4.2 Comparative cost consequences—other medical costs**

- A study has been conducted to evaluate the cost-effectiveness of VPZ in comparison to PPIs for the treatment of GERD patients in China.
- Other medical costs associated with EE treatment are listed in Supplementary Table 4.
- Treatment with VPZ resulted in 4.35 quality adjusted life years (QALYs) at a total cost of USD 1,354 over 5 years. Compared with the PPI group, treating EE with VPZ was associated with 0.02 QALYs gained and a cost saving of USD 943. Thus, VPZ should be considered as the dominant treatment option. The model results were deemed robust in sensitivity analyses[49].

Supplementary Table 4 Other medical costs associated with EE

| Medical cost | Items | Cost (USD) | Notes |
| --- | --- | --- | --- |
| Outpatient visit | Visit during the healing phase | 15 | Twice a month |
|  | Visit during the maintenance phase | 7 | Once a month |
| Endoscopy | For diagnosis | 84 | Once |
| 24-hour pH monitoring | For patients who were unhealed after 8-week treatment | 116 | Once |

## **4.3 Comparative cost consequences—non-medical costs**

- Neither VPZ nor TPZ retrieved evidence.

**5. Knowledge about intervention (Absolute Criterion)**

## **5.1 Quality of evidence**

- All clinical trials included in this study were randomized trials, which were conducted in accordance with the Good Clinical Practices (GCP) guidelines of the International Council for Harmonization of Technical Requirements for Pharmaceuticals for Human Use (ICH), the principles of the Declaration of Helsinki and the local laws of the countries involved.
- It should be noted that there is still a lack of direct comparisons between VPZ and TPZ. The control groups in clinical trials were placebo or PPIs. This can only provide evidence for an indirect comparison. For this reason, all systematic reviews and meta-analyses can only provide results for comparisons with PPIs.
- Given the practical needs of decision-making, we used only the pharmacoeconomic studies conducted in China. A Markov model was developed to predict the effectiveness and cost of VPZ versus other PPIs for treating GERD in China. This model is well studied for modelling the progression of chronic diseases and has been widely used in economic evaluations of GERD treatment. The model included an acute treatment phase and a maintenance phase, reflecting the general treatment pattern of all GERD patients as well as GERD patients with moderate to severe disease[49].
- Evidence for absolute indicators includes systematic reviews and meta-analyses, evidence-based clinical practice guidelines, expert consensus, epidemiological studies, etc. All evidence is reviewed by clinical experts.

## **5.2 Expert consensus/clinical practice guidelines**

- Chinese consensus on multidisciplinary diagnosis and treatment of GERD 2022, recommendations for drug therapy are as follows: PPIs and P-CABs are the first-line treatment for GERD, relieving symptoms and complications in most people with GERD[50, 51].
- Evidence-based clinical practice guidelines for GERD 2021 in Japan, recommendations for drug therapy are as follows: (1) PPIs and P-CABs both achieve esophageal mucosal healing in the initial treatment of mild RE. Both medications are recommended as a first-line treatment for patients with mild RE (Strong recommendation, Evidence level B, 100% agreed). (2) VPZ at 20 mg once daily for 4 weeks is proposed as the initial treatment of patients with severe RE (Weak recommendation, Evidence level C, 100% agreed). (3) P-CABs are proposed for the long-term maintenance of mild RE (Weak recommendation, Evidence level C, 86% agreed)[4].
- 2020 Seoul Consensus on the Diagnosis and Management of Gastroesophageal Reflux Disease, recommendations for drug therapy are as follows: The efficacy of P-CABs is comparable to PPIs, hence they are recommended as an initial treatment of GERD. Level of evidence: moderate. Strength of recommendation: strong[52].
- European Society of Neurogastroenterology and Motility (ESNM)/American Neurogastroenterology and Motility Society (ANMS) consensus paper: Diagnosis and management of refractory GERD 2020, recommendations for drug therapy are as follows: P-CABs taken once-daily control intragastric pH more effectively and rapidly than any standard-dose PPIs taken once daily and have potential value in refractory GERD[5].

References

[1] FASS R, BOECKXSTAENS G E, EL-SERAG H, et al. Gastro-oesophageal reflux disease. Nat Rev Dis Primers, 2021,7(1): 55.

[2] LAGERGREN J, BERGSTROM R, LINDGREN A, et al. Symptomatic gastroesophageal reflux as a risk factor for esophageal adenocarcinoma. N Engl J Med, 1999,340(11): 825-831.

[3] YADLAPATI R, GYAWALI C P, PANDOLFINO J E. AGA Clinical Practice Update on the Personalized Approach to the Evaluation and Management of GERD: Expert Review. Clin Gastroenterol Hepatol, 2022,20(5): 984-994.

[4] IWAKIRI K, FUJIWARA Y, MANABE N, et al. Evidence-based clinical practice guidelines for gastroesophageal reflux disease 2021. J Gastroenterol, 2022,57(4): 267-285.

[5] ZERBIB F, BREDENOORD A J, FASS R, et al. ESNM/ANMS consensus paper: Diagnosis and management of refractory gastro-esophageal reflux disease. Neurogastroenterol Motil, 2021,33(4): e14075.

[6] NESS-JENSEN E, SANTONI G, GOTTLIEB-VEDI E, et al. Mortality in gastro-oesophageal reflux disease in a population-based nationwide cohort study of Swedish twins. BMJ Open, 2020,10(8): e37456.

[7] SOLAYMANI-DODARAN M, LOGAN R F, WEST J, et al. Mortality associated with Barrett's esophagus and gastroesophageal reflux disease diagnoses-a population-based cohort study. Am J Gastroenterol, 2005,100(12): 2616-2621.

[8] EUSEBI L H, CIROTA G G, ZAGARI R M, et al. Global prevalence of Barrett's oesophagus and oesophageal cancer in individuals with gastro-oesophageal reflux: a systematic review and meta-analysis. Gut, 2021,70(3): 456-463.

[9] FULLARD M, KANG J Y, NEILD P, et al. Systematic review: does gastro-oesophageal reflux disease progress?. Aliment Pharmacol Ther, 2006,24(1): 33-45.

[10] MALFERTHEINER P, NOCON M, VIETH M, et al. Evolution of gastro-oesophageal reflux disease over 5 years under routine medical care--the ProGERD study. Aliment Pharmacol Ther, 2012,35(1): 154-164.

[11] WANG R, YAN X, MA X Q, et al. Burden of gastroesophageal reflux disease in Shanghai, China. Dig Liver Dis, 2009,41(2): 110-115.

[12] NIRWAN J S, HASAN S S, BABAR Z U, et al. Global Prevalence and Risk Factors of Gastro-oesophageal Reflux Disease (GORD): Systematic Review with Meta-analysis. Sci Rep, 2020,10(1): 5814.

[13] DIRAC M A, SAFIRI S, TSOI D, et al. The global, regional, and national burden of gastro-oesophageal reflux disease in 195 countries and territories, 1990-2017: a systematic analysis for the Global Burden of Disease Study 2017. Lancet Gastroenterol Hepatol, 2020,5(6): 561-581.

[14] EUSEBI L H, RATNAKUMARAN R, YUAN Y, et al. Global prevalence of, and risk factors for, gastro-oesophageal reflux symptoms: a meta-analysis. Gut, 2018,67(3): 430-440.

[15] CARLSSON R, DENT J, WATTS R, et al. Gastro-oesophageal reflux disease in primary care: an international study of different treatment strategies with omeprazole. International GORD Study Group. Eur J Gastroenterol Hepatol, 1998,10(2): 119-124.

[16] FASS R, SIFRIM D. Management of heartburn not responding to proton pump inhibitors. Gut, 2009,58(2): 295-309.

[17] HERSHCOVICI T, FASS R. Management of gastroesophageal reflux disease that does not respond well to proton pump inhibitors. Curr Opin Gastroenterol, 2010,26(4): 367-378.

[18] INADOMI J M, MCINTYRE L, BERNARD L, et al. Step-down from multiple- to single-dose proton pump inhibitors (PPIs): a prospective study of patients with heartburn or acid regurgitation completely relieved with PPIs. Am J Gastroenterol, 2003,98(9): 1940-1944.

[19] ICHIKAWA H, SUGIMOTO M, SUGIMOTO K, et al. Rapid metabolizer genotype of CYP2C19 is a risk factor of being refractory to proton pump inhibitor therapy for reflux esophagitis. J Gastroenterol Hepatol, 2016,31(4): 716-726.

[20] MARET-OUDA J, MARKAR S R, LAGERGREN J. Gastroesophageal Reflux Disease: A Review. JAMA, 2020,324(24): 2536-2547.

[21] ASHIDA K, SAKURAI Y, NISHIMURA A, et al. Randomised clinical trial: a dose-ranging study of vonoprazan, a novel potassium-competitive acid blocker, vs. lansoprazole for the treatment of erosive oesophagitis. Aliment Pharmacol Ther, 2015,42(6): 685-695.

[22] ASHIDA K, SAKURAI Y, HORI T, et al. Randomised clinical trial: vonoprazan, a novel potassium-competitive acid blocker, vs. lansoprazole for the healing of erosive oesophagitis. Aliment Pharmacol Ther, 2016,43(2): 240-251.

[23] XIAO Y, ZHANG S, DAI N, et al. Phase III, randomised, double-blind, multicentre study to evaluate the efficacy and safety of vonoprazan compared with lansoprazole in Asian patients with erosive oesophagitis. Gut, 2020,69(2): 224-230.

[24] LEE K J, SON B K, KIM G H, et al. Randomised phase 3 trial: tegoprazan, a novel potassium-competitive acid blocker, vs. esomeprazole in patients with erosive oesophagitis. Aliment Pharmacol Ther, 2019,49(7): 864-872.

[25] TAKEUCHI T, FURUTA T, FUJIWARA Y, et al. Randomised trial of acid inhibition by vonoprazan 10/20 mg once daily vs rabeprazole 10/20 mg twice daily in healthy Japanese volunteers (SAMURAI pH study). Aliment Pharmacol Ther, 2020,51(5): 534-543.

[26] SAKURAI Y, MORI Y, OKAMOTO H, et al. Acid-inhibitory effects of vonoprazan 20 mg compared with esomeprazole 20 mg or rabeprazole 10 mg in healthy adult male subjects--a randomised open-label cross-over study. Aliment Pharmacol Ther, 2015,42(6): 719-730.

[27] LAINE L, SHARMA P, MULFORD D J, et al. Pharmacodynamics and Pharmacokinetics of the Potassium-Competitive Acid Blocker Vonoprazan and the Proton Pump Inhibitor Lansoprazole in US Subjects. Am J Gastroenterol, 2022,117(7): 1158-1161.

[28] YANG E, KIM S, KIM B, et al. Night-time gastric acid suppression by tegoprazan compared to vonoprazan or esomeprazole. Br J Clin Pharmacol, 2022,88(7): 3288-3296.

[29] HAN S, CHOI H Y, KIM Y H, et al. Comparison of Pharmacodynamics between Tegoprazan and Dexlansoprazole Regarding Nocturnal Acid Breakthrough: A Randomized Crossover Study. Gut Liver, 2023,17(1): 92-99.

[30] CHENG Y, LIU J, TAN X, et al. Direct Comparison of the Efficacy and Safety of Vonoprazan Versus Proton-Pump Inhibitors for Gastroesophageal Reflux Disease: A Systematic Review and Meta-Analysis. Dig Dis Sci, 2021,66(1): 19-28.

[31] XU W, BAI Z, SHANG Y, et al. Incidence and type of adverse events in patients taking vonoprazan: A systematic review and meta-analysis. Therap Adv Gastroenterol, 2023,16: 1108350834.

[32] GONG H, HAN D, LIU S, et al. Adverse events of vonoprazan in the treatments of acid-related diseases: a systematic review and meta-analysis. Rev Esp Enferm Dig, 2023,115(6): 294-300.

[33] Gotoh Y, Ishibashi E, Honda S, et al. Efficacy of vonoprazan for initial and maintenance therapy in reflux esophagitis, nonerosive esophagitis, and proton pump inhibitor-resistant gastroesophageal reflux disease. Medicine (Baltimore). 2020;99(11):e19520.

[34] CHO Y K, CHOI M G, CHOI S C, et al. Randomised clinical trial: tegoprazan, a novel potassium-competitive acid blocker, or lansoprazole in the treatment of gastric ulcer. Aliment Pharmacol Ther, 2020,52(5): 789-797.

[35] KIM J S, SEO S I, KANG S H, et al. Effects of Tegoprazan Versus Esomeprazole on Nighttime Heartburn and Sleep Quality in Gastroesophageal Reflux Disease: A Multicenter Double-blind Randomized Controlled Trial. J Neurogastroenterol Motil, 2023,29(1): 58-64.

[36] CHO Y K, KIM J H, KIM H S, et al. Randomised clinical trial: comparison of tegoprazan and lansoprazole as maintenance therapy for healed mild erosive oesophagitis. Aliment Pharmacol Ther, 2023,57(1): 72-80.

[37] TACK J, VLADIMIROV B, HORNY I, et al. Randomized clinical trial: A double-blind, proof-of-concept, phase 2 study evaluating the efficacy and safety of vonoprazan 20 or 40 mg versus esomeprazole 40 mg in patients with symptomatic gastro-esophageal reflux disease and partial response to a healing dose of a proton-pump inhibitor. Neurogastroenterol Motil, 2023,35(1): e14468.

[38] OSHIMA T, ARAI E, TAKI M, et al. Randomised clinical trial: vonoprazan versus lansoprazole for the initial relief of heartburn in patients with erosive oesophagitis. Aliment Pharmacol Ther, 2019,49(2): 140-146.

[39] KINOSHITA Y, SAKURAI Y, TAKABAYASHI N, et al. Efficacy and Safety of Vonoprazan in Patients With Nonerosive Gastroesophageal Reflux Disease: A Randomized, Placebo-Controlled, Phase 3 Study. Clin Transl Gastroenterol, 2019,10(11): e101.

[40] KIM S H, CHO K B, CHUN H J, et al. Randomised clinical trial: comparison of tegoprazan and placebo in non-erosive reflux disease. Aliment Pharmacol Ther, 2021,54(4): 402-411.

[41] OKANOBU H, KOHNO T, BODA K, et al. Long-Term Clinical Course after Successful Initial Treatment in Patients with Mild Erosive Esophagitis: A Prospective Follow-Up Study. Digestion, 2023,104(4): 283-290.

[42] TANABE T, HOSHINO S, KAWAMI N, et al. Efficacy of long-term maintenance therapy with 10-mg vonoprazan for proton pump inhibitor-resistant reflux esophagitis. Esophagus, 2019,16(4): 377-381.

[43] ASHIDA K, IWAKIRI K, HIRAMATSU N, et al. Maintenance for healed erosive esophagitis: Phase III comparison of vonoprazan with lansoprazole. World J Gastroenterol, 2018,24(14): 1550-1561.

[44] UMEZAWA M, KAWAMI N, HOSHINO S, et al. Efficacy of On-Demand Therapy Using 20-mg Vonoprazan for Mild Reflux Esophagitis. Digestion, 2018,97(4): 309-315.

[45] MIZUNO H, NISHINO M, YAMADA K, et al. Efficacy of Vonoprazan for 48-Week Maintenance Therapy of Patients with Healed Reflux Esophagitis. Digestion, 2020,101(4): 411-421.

[46] KAGAMI T, SAHARA S, ICHIKAWA H, et al. Potent acid inhibition by vonoprazan in comparison with esomeprazole, with reference to CYP2C19 genotype. Aliment Pharmacol Ther, 2016,43(10): 1048-1059.

[47] HOSHINO S, KAWAMI N, TAKENOUCHI N, et al. Efficacy of Vonoprazan for Proton Pump Inhibitor-Resistant Reflux Esophagitis. Digestion, 2017,95(2): 156-161.

[48] ZHANG M, XIAO Y, CHEN M. The role of vonoprazan in patients with erosive esophagitis. Therap Adv Gastroenterol, 2022,15: 1098305599.

[49] WANG Z, SUN R, SHENG Y, et al. Cost-effectiveness analysis of vonoprazan versus proton pump inhibitors in the treatment of reflux esophagitis in China. Ann Transl Med, 2022,10(8): 480.

[50] China International Exchange and Promotive Association for Medical and Health Care Gastroesophageal Reflux Disease Society. Chinese consensus on multidisciplinary diagnosis and treatment of gastroesophageal reflux disease 2022(1). Chinese Journal of Gastroesophageal Reflux Disease (Electronic Edition), 2022,09(3): 112-146.

[51] China International Exchange and Promotive Association for Medical and Health Care Gastroesophageal Reflux Disease Society. Chinese consensus on multidisciplinary diagnosis and treatment of gastroesophageal reflux disease 2022(2). Chinese Journal of Gastroesophageal Reflux Disease (Electronic Edition), 2022,09(2): 51-86.

[52] JUNG H K, TAE C H, SONG K H, et al. 2020 Seoul Consensus on the Diagnosis and Management of Gastroesophageal Reflux Disease. J Neurogastroenterol Motil, 2021,27(4): 453-481.

**Search formula**

**PubMed**

| Domains | PubMed |
| --- | --- |
| Need for intervention | #1 "gastroesophageal reflux"[MeSH Terms] OR ("gastroesophageal"[All Fields] AND "reflux"[All Fields]) OR "gastroesophageal reflux"[All Fields] OR "gerd"[All Fields] #2 (("Epidemiology"[Mesh]) OR "epidemiology" [Subheading]) OR (((((((((((epidemics[Title/Abstract]) OR frequency[Title/Abstract]) OR surveillance[Title/Abstract]) OR morbidity[Title/Abstract]) OR occurrence[Title/Abstract]) OR outbreaks[Title/Abstract]) OR prevalence[Title/Abstract]) OR endemics[Title/Abstract]) OR incidence[Title/Abstract]) OR mortality[Title/Abstract])) #3 #1 AND #2 |
| Comparative outcomes of intervention | #1 "gastroesophageal reflux"[MeSH Terms] OR ("gastroesophageal"[All Fields] AND "reflux"[All Fields]) OR "gastroesophageal reflux"[All Fields] OR "gerd"[All Fields] #2 "1-(5-(2-fluorophenyl)-1-(pyridin-3-ylsulfonyl)-1H-pyrrol-3-yl)-N-methylmethanamine"[Supplementary Concept] OR "1-(5-(2-fluorophenyl)-1-(pyridin-3-ylsulfonyl)-1H-pyrrol-3-yl)-N-methylmethanamine"[All Fields] OR "vonoprazan"[All Fields]AND (clinicaltrial[Filter] OR meta-analysis[Filter] OR randomizedcontrolledtrial[Filter] OR systematicreview[Filter]) #3 ("tegoprazan"[Supplementary Concept] OR "tegoprazan"[All Fields]) AND (clinicaltrial[Filter] OR meta-analysis[Filter] OR randomizedcontrolledtrial[Filter] OR systematicreview[Filter]) #4 #1 AND #2 #5 #1 AND #3 #6 #4 OR #5 |
| Type of benefit of intervention | #1 "gastroesophageal reflux"[MeSH Terms] OR ("gastroesophageal"[All Fields] AND "reflux"[All Fields]) OR "gastroesophageal reflux"[All Fields] OR "gerd"[All Fields] #2 "1-(5-(2-fluorophenyl)-1-(pyridin-3-ylsulfonyl)-1H-pyrrol-3-yl)-N-methylmethanamine"[Supplementary Concept] OR "1-(5-(2-fluorophenyl)-1-(pyridin-3-ylsulfonyl)-1H-pyrrol-3-yl)-N-methylmethanamine"[All Fields] OR "vonoprazan"[All Fields] #3 ("tegoprazan"[Supplementary Concept] OR "tegoprazan"[All Fields]) #4 ("Patient Reported Outcome Measures"[Mesh]) OR ((((((((Patient Reported Outcomes[Title/Abstract]) OR Outcome, Patient Reported[Title/Abstract]) OR Outcomes, Patient Reported[Title/Abstract]) OR Reported Outcome, Patient[Title/Abstract]) OR Reported Outcomes, Patient[Title/Abstract]) OR Patient Reported Outcome[Title/Abstract]) OR PRO[Title/Abstract])) #5 #1 AND #2 AND #4 #6 #1 AND #3 AND #4 #7 #5 OR #6 |
| Economic consequences of intervention | #1 ("Economics"[Mesh]) OR (((((((((((((((((((((((((((((((((((Consumer Price Index[Title/Abstract]) OR Consumer Price Index[Title/Abstract]) OR Index, Consumer Price[Title/Abstract]) OR Indices, Consumer Price[Title/Abstract]) OR Consumption[Title/Abstract]) OR Cost of Living[Title/Abstract]) OR Living Cost[Title/Abstract]) OR Living Costs[Title/Abstract]) OR Easterlin Hypothesis[Title/Abstract]) OR Hypothesis, Easterlin[Title/Abstract]) OR Economic Condition*[Title/Abstract]) OR Condition*, Economic[Title/Abstract]) OR Economic Policies[Title/Abstract]) OR Policies, Economic[Title/Abstract]) OR Policy, Economic[Title/Abstract]) OR Economic Policy[Title/Abstract]) OR Factor*, Economic[Title/Abstract]) OR Economic Factor*[Title/Abstract]) OR Home Economics[Title/Abstract]) OR Economics, Home[Title/Abstract]) OR Household Consumption*[Title/Abstract]) OR Consumption, Household[Title/Abstract]) OR Macroeconomic Factors[Title/Abstract]) OR Factor*, Macroeconomic[Title/Abstract]) OR Microeconomic Factors[Title/Abstract]) OR Factor*, Microeconomic[Title/Abstract]) OR Production[Title/Abstract]) OR Remittance*[Title/Abstract]) OR Utility Theor*[Title/Abstract]) OR Theor*, Utility[Title/Abstract]) OR Capital[Title/Abstract]) OR cost[Title/Abstract]) OR costs[Title/Abstract]) OR econom*[Title/Abstract])) #2 "1-(5-(2-fluorophenyl)-1-(pyridin-3-ylsulfonyl)-1H-pyrrol-3-yl)-N-methylmethanamine"[Supplementary Concept] OR "1-(5-(2-fluorophenyl)-1-(pyridin-3-ylsulfonyl)-1H-pyrrol-3-yl)-N-methylmethanamine"[All Fields] OR "vonoprazan"[All Fields] #3 ("tegoprazan"[Supplementary Concept] OR "tegoprazan"[All Fields]) #4 #1 AND #2 #5 #1 AND #3 #6 #4 OR #5 |
| Knowledge about intervention | #1 ("Practice Guideline" [Publication Type]) OR (((((Clinical Practice Guideline*[Title/Abstract]) OR guideline*[Title/Abstract]) OR recommendations[Title/Abstract]) OR clinical practice [Title/Abstract])) #2 ("Consensus"[Mesh]) OR ((((Consensus Development[Title/Abstract]) OR Development, Consensus[Title/Abstract]) OR expert consensus[Title/Abstract])) #3 #1 OR #2  #4 "gastroesophageal reflux"[MeSH Terms] OR ("gastroesophageal"[All Fields] AND "reflux"[All Fields]) OR "gastroesophageal reflux"[All Fields] OR "gerd"[All Fields] #5 #3 AND #4 |

**The Cochrane Library**

| Domains | The Cochrane Library |
| --- | --- |
| Need for intervention | #1 "gastroesophageal reflux"[MeSH Terms] OR ("gastroesophageal"[All Fields] AND "reflux"[All Fields]) OR "gastroesophageal reflux"[All Fields] OR "gerd"[All Fields] #2 (("Epidemiology"[Mesh]) OR "epidemiology" [Subheading]) OR (((((((((((epidemics[Title/Abstract]) OR frequency[Title/Abstract]) OR surveillance[Title/Abstract]) OR morbidity[Title/Abstract]) OR occurrence[Title/Abstract]) OR outbreaks[Title/Abstract]) OR prevalence[Title/Abstract]) OR endemics[Title/Abstract]) OR incidence[Title/Abstract]) OR mortality[Title/Abstract])) #3 #1 AND #2 |
| Comparative outcomes of intervention | #1 "gastroesophageal reflux"[MeSH Terms] OR ("gastroesophageal"[All Fields] AND "reflux"[All Fields]) OR "gastroesophageal reflux"[All Fields] OR "gerd"[All Fields] #2 "1-(5-(2-fluorophenyl)-1-(pyridin-3-ylsulfonyl)-1H-pyrrol-3-yl)-N-methylmethanamine"[Supplementary Concept] OR "1-(5-(2-fluorophenyl)-1-(pyridin-3-ylsulfonyl)-1H-pyrrol-3-yl)-N-methylmethanamine"[All Fields] OR "vonoprazan"[All Fields]AND (clinicaltrial[Filter] OR meta-analysis[Filter] OR randomizedcontrolledtrial[Filter] OR systematicreview[Filter]) #3 ("tegoprazan"[Supplementary Concept] OR "tegoprazan"[All Fields]) AND (clinicaltrial[Filter] OR meta-analysis[Filter] OR randomizedcontrolledtrial[Filter] OR systematicreview[Filter]) #4 #1 AND #2 #5 #1 AND #3 #6 #4 OR #5 |
| Type of benefit of intervention | #1 "gastroesophageal reflux"[MeSH Terms] OR ("gastroesophageal"[All Fields] AND "reflux"[All Fields]) OR "gastroesophageal reflux"[All Fields] OR "gerd"[All Fields] #2 "1-(5-(2-fluorophenyl)-1-(pyridin-3-ylsulfonyl)-1H-pyrrol-3-yl)-N-methylmethanamine"[Supplementary Concept] OR "1-(5-(2-fluorophenyl)-1-(pyridin-3-ylsulfonyl)-1H-pyrrol-3-yl)-N-methylmethanamine"[All Fields] OR "vonoprazan"[All Fields] #3 ("tegoprazan"[Supplementary Concept] OR "tegoprazan"[All Fields]) #4 ("Patient Reported Outcome Measures"[Mesh]) OR ((((((((Patient Reported Outcomes[Title/Abstract]) OR Outcome, Patient Reported[Title/Abstract]) OR Outcomes, Patient Reported[Title/Abstract]) OR Reported Outcome, Patient[Title/Abstract]) OR Reported Outcomes, Patient[Title/Abstract]) OR Patient Reported Outcome[Title/Abstract]) OR PRO[Title/Abstract])) #5 #1 AND #2 AND #4 #6 #1 AND #3 AND #4 #7 #5 OR #6 |
| Economic consequences of intervention | #1 ("Economics"[Mesh]) OR (((((((((((((((((((((((((((((((((((Consumer Price Index[Title/Abstract]) OR Consumer Price Index[Title/Abstract]) OR Index, Consumer Price[Title/Abstract]) OR Indices, Consumer Price[Title/Abstract]) OR Consumption[Title/Abstract]) OR Cost of Living[Title/Abstract]) OR Living Cost[Title/Abstract]) OR Living Costs[Title/Abstract]) OR Easterlin Hypothesis[Title/Abstract]) OR Hypothesis, Easterlin[Title/Abstract]) OR Economic Condition*[Title/Abstract]) OR Condition*, Economic[Title/Abstract]) OR Economic Policies[Title/Abstract]) OR Policies, Economic[Title/Abstract]) OR Policy, Economic[Title/Abstract]) OR Economic Policy[Title/Abstract]) OR Factor*, Economic[Title/Abstract]) OR Economic Factor*[Title/Abstract]) OR Home Economics[Title/Abstract]) OR Economics, Home[Title/Abstract]) OR Household Consumption*[Title/Abstract]) OR Consumption, Household[Title/Abstract]) OR Macroeconomic Factors[Title/Abstract]) OR Factor*, Macroeconomic[Title/Abstract]) OR Microeconomic Factors[Title/Abstract]) OR Factor*, Microeconomic[Title/Abstract]) OR Production[Title/Abstract]) OR Remittance*[Title/Abstract]) OR Utility Theor*[Title/Abstract]) OR Theor*, Utility[Title/Abstract]) OR Capital[Title/Abstract]) OR cost[Title/Abstract]) OR costs[Title/Abstract]) OR econom*[Title/Abstract])) #2 "1-(5-(2-fluorophenyl)-1-(pyridin-3-ylsulfonyl)-1H-pyrrol-3-yl)-N-methylmethanamine"[Supplementary Concept] OR "1-(5-(2-fluorophenyl)-1-(pyridin-3-ylsulfonyl)-1H-pyrrol-3-yl)-N-methylmethanamine"[All Fields] OR "vonoprazan"[All Fields] #3 ("tegoprazan"[Supplementary Concept] OR "tegoprazan"[All Fields]) #4 #1 AND #2 #5 #1 AND #3 #6 #4 OR #5 |
| Knowledge about intervention | #1 ("Practice Guideline" [Publication Type]) OR (((((Clinical Practice Guideline*[Title/Abstract]) OR guideline*[Title/Abstract]) OR recommendations[Title/Abstract]) OR clinical practice [Title/Abstract])) #2 ("Consensus"[Mesh]) OR ((((Consensus Development[Title/Abstract]) OR Development, Consensus[Title/Abstract]) OR expert consensus[Title/Abstract])) #3 #1 OR #2  #4 "gastroesophageal reflux"[MeSH Terms] OR ("gastroesophageal"[All Fields] AND "reflux"[All Fields]) OR "gastroesophageal reflux"[All Fields] OR "gerd"[All Fields] #5 #3 AND #4 |

**Embase**

| Domains | Embase |
| --- | --- |
| Need for intervention | #1 'gastroesophageal reflux'/exp OR 'gastroesophageal reflux' OR (gastroesophageal AND reflux) #2 'epidemics'/exp OR 'epidemics' OR 'frequency'/exp OR 'frequency' OR 'surveillance'/exp OR 'surveillance' OR 'morbidity'/exp OR 'morbidity' OR 'occurrence' OR 'outbreaks' OR 'prevalence'/exp OR 'prevalence' OR 'endemics' OR 'incidence'/exp OR 'incidence' OR 'mortality':ab,ti #3 #1 AND #2 |
| Comparative outcomes of intervention | #1 'gastroesophageal reflux'/exp OR 'gastroesophageal reflux' OR (gastroesophageal AND reflux) #2 ('vonoprazan'/exp OR vonoprazan) AND ([cochrane review]/lim OR [controlled clinical trial]/lim OR [systematic review]/lim OR [randomized controlled trial]/lim OR [meta analysis]/lim) #3 ('tegoprazan'/exp OR tegoprazan) AND ([cochrane review]/lim OR [controlled clinical trial]/lim OR [systematic review]/lim OR [randomized controlled trial]/lim OR [meta analysis]/lim) #4 #1 AND #2 #5 #1 AND #3 #6 #4 OR #5 |
| Type of benefit of intervention | #1 'gastroesophageal reflux'/exp OR 'gastroesophageal reflux' OR (gastroesophageal AND reflux) #2 'vonoprazan'/exp OR vonoprazan #3 'tegoprazan'/exp OR tegoprazan #4 'patient reported outcome measures'/exp OR 'patient reported outcome measures' OR 'patient reported outcomes'/exp OR 'patient reported outcomes' OR 'outcome, patient reported' OR 'outcomes, patient reported' OR 'reported outcome, patient' OR 'reported outcomes, patient' OR 'patient reported outcome'/exp OR 'patient reported outcome' OR 'pro' OR 'patient reported outcome*':ab,ti #5 #1 AND #2 AND #4 #6 #1 AND #3 AND #4 #7 #5 OR #6 |
| Economic consequences of intervention | #1 'economics'/exp OR 'consumer price index'/exp OR 'consumer price index' OR 'consumer price indices' OR 'index, consumer price' OR 'indices, consumer price' OR 'consumption'/exp OR 'consumption' OR 'cost of living'/exp OR 'cost of living' OR 'living cost' OR 'living costs' OR 'easterlin hypothesis'/exp OR 'easterlin hypothesis' OR 'hypothesis, easterlin' OR 'economic condition*' OR 'condition*, economic' OR 'economic policies' OR 'policies, economic' OR 'policy, economic' OR 'economic policy'/exp OR 'economic policy' OR 'factor*, economic' OR 'economic factor*' OR 'home economics'/exp OR 'home economics' OR 'economics, home' OR 'household consumption*' OR 'consumption, household' OR 'macroeconomic factors'/exp OR 'macroeconomic factors' OR 'factor*, macroeconomic' OR 'microeconomic factors'/exp OR 'microeconomic factors' OR 'factor*, microeconomic' OR 'production'/exp OR 'production' OR 'remittance*' OR 'utility theor*' OR 'theor*, utility' OR 'capital'/exp OR 'capital' OR 'cost'/exp OR 'cost' OR 'costs' OR 'econom*':ab,ti #2 'vonoprazan'/exp OR vonoprazan #3 'tegoprazan'/exp OR tegoprazan #4 #1 AND #2 #5 #1 AND #3 #6 #4 OR #5 |
| Knowledge about intervention | #1 'practice guideline'/exp OR 'clinical practice guideline*' OR 'guideline*' OR 'recommendations'/exp OR 'recommendations' OR 'clinical practice':ab,ti #2 'consensus'/exp OR 'consensus development'/exp OR 'consensus development' OR 'development, consensus' OR 'expert consensus':ab,ti #3 #1 OR #2 #4 'gastroesophageal reflux'/exp OR 'gastroesophageal reflux' OR (gastroesophageal AND reflux) #5 #3 AND #4 |

**China Biology Medicine disc**

| Domains | China Biology Medicine disc |
| --- | --- |
| Need for intervention | #1 ("gastroesophageal reflux"[All Fields] OR "Gastric Acid Reflux"[All Fields] OR "Gastric Acid Reflux Disease"[All Fields] OR "Gastro-Esophageal Reflux Disease"[All Fields] OR "Gastro Esophageal Reflux Disease"[All Fields] OR "Gastro-Esophageal Reflux Diseases"[All Fields] OR "Gastro-oesophageal Reflux"[All Fields] OR "Gastro oesophageal Reflux"[All Fields] OR "Gastroesophageal Reflux Disease"[All Fields] OR "GERD"[All Fields] OR "Esophageal Reflux"[All Fields] OR "Gastro-Esophageal Reflux"[All Fields] OR "Gastro Esophageal Reflux"[All Fields] #2 ("Epidemiology"[All Fields] OR "Social Epidemiology"[All Fields] OR "Social Epidemiologies"[All Fields] OR "surveillance"[All Fields] OR ("morbidity"[All Fields] OR "Morbidities"[All Fields] OR ("prevalence"[All Fields] OR "Prevalences"[All Fields] OR "Period Prevalence"[All Fields] OR "Period Prevalences"[All Fields] OR "Point Prevalence"[All Fields] OR "Point Prevalences"[All Fields] OR ("incidence"[All Fields] OR "Incidences"[All Fields] OR "Scondary Attack Rate"[All Fields] OR "Scondary Attack Rates"[All Fields] OR "Incidence Proportion"[All Fields] OR "Incidence Proportions"[All Fields] OR "Attack Rate"[All Fields] OR "Attack Rates"[All Fields] OR "Cumulative Incidence"[All Fields] OR "Cumulative Incidences"[All Fields] OR "Incidence Rate"[All Fields] OR "Incidence Rates"[All Fields] OR "Person-time Rate"[All Fields] OR "Person time Rate"[All Fields] OR "Person-time Rates"[All Fields] OR ("mortality"[All Fields] OR "Mortality Determinant"[All Fields] OR "Differential Mortality"[All Fields] OR "Differential Mortalities"[All Fields] OR "Age Specific Death Rate"[All Fields] OR "Age-Specific Death Rate"[All Fields] OR "Age-Specific Death Rates"[All Fields] OR "Mortalities"[All Fields] OR "Case Fatality Rate"[All Fields] OR "Case Fatality Rates"[All Fields] OR "CFR Case Fatality Rate"[All Fields] OR "Crude Death Rate"[All Fields] OR "Crude Death Rates"[All Fields] OR "Crude Mortality Rate"[All Fields] OR "Crude Mortality Rates"[All Fields] OR "Death Rate"[All Fields] OR "Death Rates"[All Fields] OR "Mortality Rate"[All Fields] OR "Mortality Rates"[All Fields] OR "Excess Mortality"[All Fields] OR "Excess Mortalities"[All Fields] OR "Mortality Decline"[All Fields] OR "Mortality Declines"[All Fields] OR "Mortality Determinants"[All Fields] #3 #1 AND #2 |
| Comparative outcomes of intervention | #1 ("gastroesophageal reflux"[All Fields] OR "Gastric Acid Reflux"[All Fields] OR "Gastric Acid Reflux Disease"[All Fields] OR "Gastro-Esophageal Reflux Disease"[All Fields] OR "Gastro Esophageal Reflux Disease"[All Fields] OR "Gastro-Esophageal Reflux Diseases"[All Fields] OR "Gastro-oesophageal Reflux"[All Fields] OR "Gastro oesophageal Reflux"[All Fields] OR "Gastroesophageal Reflux Disease"[All Fields] OR "GERD"[All Fields] OR "Esophageal Reflux"[All Fields] OR "Gastro-Esophageal Reflux"[All Fields] OR "Gastro Esophageal Reflux"[All Fields] #2 "vonoprazan"[All Fields] AND (clinicaltrial[Filter] OR meta-analysis[Filter] OR randomizedcontrolledtrial[Filter] OR systematicreview[Filter]) #3 "tegoprazan"[All Fields] AND (clinicaltrial[Filter] OR meta-analysis[Filter] OR randomizedcontrolledtrial[Filter] OR systematicreview[Filter]) #4 #1 AND #2 #5 #1 AND #3 #6 #4 OR #5 |
| Type of benefit of intervention | #1 ("gastroesophageal reflux"[All Fields] OR "Gastric Acid Reflux"[All Fields] OR "Gastric Acid Reflux Disease"[All Fields] OR "Gastro-Esophageal Reflux Disease"[All Fields] OR "Gastro Esophageal Reflux Disease"[All Fields] OR "Gastro-Esophageal Reflux Diseases"[All Fields] OR "Gastro-oesophageal Reflux"[All Fields] OR "Gastro oesophageal Reflux"[All Fields] OR "Gastroesophageal Reflux Disease"[All Fields] OR "GERD"[All Fields] OR "Esophageal Reflux"[All Fields] OR "Gastro-Esophageal Reflux"[All Fields] OR "Gastro Esophageal Reflux"[All Fields] #2 "vonoprazan"[All Fields] #3 "tegoprazan"[All Fields] #4 ("Patient Reported Outcome Measures"[All Fields] OR "Patient Reported Outcome Measure"[All Fields] OR "Patient Reported Outcomes"[All Fields] OR "Patient Reported Outcome"[All Fields] OR "Patient-Reported Outcome"[All Fields] OR "Patient-Reported Outcomes"[All Fields] #5 #1 AND #2 AND #4 #6 #1 AND #3 AND #4 #7 #5 OR #6 |
| Economic consequences of intervention | #1 ("Economics"[All Fields] OR "Consumption"[All Fields] OR "Cost of Living"[All Fields] OR "Living Cost"[All Fields] OR "Living Costs"[All Fields] OR "Easterlin Hypothesis"[All Fields] OR "Economic Conditions"[All Fields] OR "Economic Condition"[All Fields] OR "Economic Policies"[All Fields] OR "Economic Policy"[All Fields] OR "Home Economics"[All Fields] OR "Household Consumption"[All Fields] OR "Household Consumptions"[All Fields] OR "Macroeconomic Factors"[All Fields] OR "Macroeconomic Factor"[All Fields] OR "Microeconomic Factors"[All Fields] OR "Microeconomic Factor"[All Fields] OR "Production"[All Fields] OR "Remittances"[All Fields] OR "Remittance"[All Fields] OR "Utility Theory"[All Fields] OR "Utility Theories"[All Fields] OR "Capital"[All Fields] OR "Consumer Price Index"[All Fields] OR "Consumer Price Indices"[All Fields] #2 "vonoprazan"[All Fields] #3 "tegoprazan"[All Fields] #4 #1 AND #2 #5 #1 AND #3 #6 #4 OR #5 |
| Knowledge about intervention | #1 ("Clinical Practice Guideline"[All Fields] OR "Practice Guideline"[All Fields] OR "Clinical Guidelines"[All Fields]  #2 ("Consensus"[All Fields] OR "Consensus Development"[All Fields] #3 #1 OR #2 #4 ("gastroesophageal reflux"[All Fields] OR "Gastric Acid Reflux"[All Fields] OR "Gastric Acid Reflux Disease"[All Fields] OR "Gastro-Esophageal Reflux Disease"[All Fields] OR "Gastro Esophageal Reflux Disease"[All Fields] OR "Gastro-Esophageal Reflux Diseases"[All Fields] OR "Gastro-oesophageal Reflux"[All Fields] OR "Gastro oesophageal Reflux"[All Fields] OR "Gastroesophageal Reflux Disease"[All Fields] OR "GERD"[All Fields] OR "Esophageal Reflux"[All Fields] OR "Gastro-Esophageal Reflux"[All Fields] OR "Gastro Esophageal Reflux"[All Fields] #5 #3 AND #4 |

1. *Correspondence:

   Zhanjun Dong

   dzjhbgh@126.com

   Hebei General Hospital, Shijiazhuang, Hebei Province, China

   ^2^ Hebei Key Laboratory of Clinical Pharmacy, Shijiazhuang, Hebei Province, China [↑](#footnote-ref-0)
